# More Than 200 Genes Required for Methane Formation from H_2_ and CO_2_ and Energy Conservation Are Present in *Methanothermobacter marburgensis* and *Methanothermobacter thermautotrophicus*

**DOI:** 10.1155/2011/973848

**Published:** 2011-04-27

**Authors:** Anne-Kristin Kaster, Meike Goenrich, Henning Seedorf, Heiko Liesegang, Antje Wollherr, Gerhard Gottschalk, Rudolf K. Thauer

**Affiliations:** ^1^Max Planck Institute for Terrestrial Microbiology, 35043 Marburg, Germany; ^2^Center for Genome Sciences and Systems Biology, Washington University School of Medicine, St. Louis MO 63108, USA; ^3^Göttingen Genomics Laboratory, Institute of Microbiology and Genetics, Georg August University, 37077 Göttingen, Germany

## Abstract

The hydrogenotrophic methanogens *Methanothermobacter marburgensis* and *Methanothermobacter thermautotrophicus* can easily be mass cultured. They have therefore been used almost exclusively to study the biochemistry of methanogenesis from H_2_ and CO_2_, and the genomes of these two model organisms have been sequenced. The close relationship of the two organisms is reflected in their genomic architecture and coding potential. Within the 1,607 protein coding sequences (CDS) in common, we identified approximately 200 CDS required for the synthesis of the enzymes, coenzymes, and prosthetic groups involved in CO_2_ reduction to methane and in coupling this process with the phosphorylation of ADP. Approximately 20 additional genes, such as those for the biosynthesis of F_430_ and methanofuran and for the posttranslational modifications of the two methyl-coenzyme M reductases, remain to be identified.

## 1. Introduction

In 1972, Zeikus and Wolfe [1] isolated *Methanothermobacter thermautotrophicus* (DSM 1053) (formerly *Methanobacterium thermoautotrophicum* strain ΔH) from sludge from the anaerobic sewage digestion plant in Urbana, Illinois, USA. This thermophile grew on H_2_ and CO_2_ as sole energy source (reaction 1) and CO_2_ as sole carbon source with doubling times of less than 5 h and to very high cell concentrations (1.5 g cells (dry mass) per L). For the first time, it became possible to obtain sufficient cell mass of a hydrogenotrophic methanogen for the purification of enzymes and coenzymes involved in CO_2_ reduction to methane. In 1978, Fuchs et al. [2] reported the isolation of *Methanothermobacter marburgensis* (DSM 2133) (formerly *Methanobacterium thermoautotrophicum* strain Marburg) from anaerobic sewage sludge in Marburg, Germany. The Marburg strain grew on H_2_ and CO_2_ even faster (doubling time less than 2 h) and to even higher cell concentrations (3 g cells (dry mass) per L) than the ΔH strain and was, therefore, subsequently used in Marburg and elsewhere for the study of methanogenesis. Most of what is presently known about the biochemistry of CO_2_ reduction to methane with H_2_ was worked out with either *M. thermautotrophicus* [3] or *M. marburgensis *[4–6]

(1) $$\begin{array}{cc} & 4{\text{H}}_{2}+{\text{CO}}_{2}\to {\text{CH}}_{4}+2{\text{H}}_{2}\text{O},\\ & \;\;\Delta G^{{}^{\circ}}=-131\;\text{kJ}\;{\text{mol}}^{-1} \end{array}\;\;\;$$

(Δ*G*° calculated for H_2_, CO_2_, and CH_4_ in the gas phase). 

The genome of *M. thermautotrophicus* (NC_000916) was one of the first genomes of Archaea to be sequenced [7] and that of *M. marburgensis* (NC_014408/CP001710) has just recently been announced [8]. This paper concentrates on the analysis of protein-coding sequences (CDS) required for the synthesis of enzymes, coenzymes, and prosthetic groups involved in CO_2_ reduction to methane with H_2_ and the coupling of this process with ATP synthesis. It provides the reader with an up-to-date picture of the molecular basis of the energy metabolism of these two hydrogenotrophic methanogens and a roadmap for defining the functional components responsible for the phenotype in other hydrogenotrophic methanogens from genome- and meta-genome data. We cite mainly the literature published after the appearance of the genome paper of Smith et al. in 1997 [7].

## 2. The Taxonomic Position of *M. marburgensis* and *M. thermautotrophicus*

All methanogens belong to the kingdom of Euryarchaeota and are presently ordered into Methanobacteriales, Methanococcales, Methanopyrales, Methanomicrobiales, Methanosarcinales, and Methanocellales ord. nov. [9, 10]. *M. marburgensis* and *M. thermautotrophicus *belong to the order of Methanobacteriales. Members of this order are very similar in their energy metabolism to the Methanococcales, Methanopyrales, and Methanomicrobiales in that their growth is, with a few exceptions, restricted to H_2_ and CO_2_ and/or to formate as energy sources. 

The methanogens of the orders of Methanobacteriales, Methanomicrobiales, Methanococcales, and Methanopyrales all lack cytochromes [11] and methanophenazine [12] as components of electron transport. In Methanococcales and Methanopyrales, several selenocysteine-containing enzymes, for example, [NiFeSe]-hydrogenases, are involved in methanogenesis from H_2_ and CO_2_ and/or from formate; the enzymes of the Methanobacteriales and Methanomicrobiales contain cysteine instead [5, 13]. Accordingly, only the growth of the Methanococcales and Methanopyrales is dependent on or stimulated by selenium.

Methanosarcinales and Methanocellales, in contrast, contain cytochromes and methanophenazine, and they do not require selenium for growth. The Methanosarcinales can grow on acetate, methanol, and/or methylamines, but only a few, for example, *Methanosarcina barkeri *and* Methanosarcina mazei,* can also grow on H_2_ and CO_2_ [14]. The growth of *Methanocella paludicola*, in contrast, appears to be restricted to H_2_ and CO_2_ and/or formate as energy sources [10, 15, 16].

The 16S rRNA of* M. marburgensis* and *M. thermautotrophicus* differs at 20 positions, resulting in a sequence identity of 98.7% [8, 17]. This suggests that the two methanogens diverged millions of years ago, as deduced from a genomic timescale based on 32 protein sequences common to 72 prokaryotic species [18].

## 3. The Phenotypes of* M. marburgensis* and *M. thermautotrophicus*

*M. marburgensis *differs from *M. thermautotrophicus* not only in the growth rate and the final cell density reached but also in the composition of the pseudomurein sacculus (galactosamine instead of glucosamine), in the size of the subunit O of DNA-dependent RNA polymerase (120 kDa instead of 96 kDa), and in the membrane-associated ATPase activity (<0.1 *μ*mol min^−1^ mg^−1^ versus 1.4 *μ*mol min^−1^ mg^−1^) [19]. Unlike *M. thermautotrophicus, M. marburgensis* contains a 4439 bp circular multicopy plasmid (pME2001 = pMTBMA4; NC_014409) [20, 21]. *M. marburgensis* is specifically infected and lysed by the phage ΨM1 [22, 23], whereas *M. thermautotrophicus* is specifically infected by the phage ΦF1 [24]. Prophage sequences have not been found in either genome sequence; however, such a sequence has been identified in the genome sequence of the closely related *Methanothermobacter wolfeii* [25].

The two *Methanothermobacter *species have in common a growth temperature optimum near 65°C and the ability to grow on H_2_ and CO_2_ as carbon and energy source, NH_3_ as nitrogen source, and H_2_S or sulfite but not sulfate as sulfur sources. *Methanothermobacter* species all require Na^+^, K^+^, Fe^2+^, Co^2+^, Ni^2+^, Zn^2+^, MoO_4_^2−^ and/or WO_4_^2−^, and possibly Ca^2+^ for growth [26, 27]. The sodium requirement is in the millimolar range. Their growth is not stimulated by the addition of organic compounds although formic acid [28], acetic acid [29], propionic acid [30], pyruvate [31], isobutyric acid, isovaleric acid, phenylacetic acid, *p*-hydroxyphenylacetic acid, indoleacetic acid [32], succinic acid [33], *δ*-aminolevulinic acid [34], methionine [35], guanine [36], and biotin [37] can be assimilated. The ability of the two *Methanothermobacter *species to assimilate formate, however, does not mean that they can grow on it as energy source, which is an ability of the related strains *M. thermautotrophicus* strain Z-245 and *Methanothermobacter wolfeii* [17, 38].

Like all members of the Methanobacteriales, *M. marburgensis* and *M. thermautotrophicus* are not motile, do not conjugate, and are devoid of heme proteins and methanophenazine and, as mentioned above, selenocysteine proteins. Accordingly, their genomes lack CDS for these functions, with a few exceptions. Each of their genomes contains CDS predicted to encode a homolog of selenophosphate synthase (SelD) (MTBMA_c04350; MTH1864) and of selenocysteine synthase (SelA) (MTBMA_c04850; MTH1914), which catalyzes the formation of Sec-tRNA^Sec^ from Ser-tRNA^Ser^ using selenophosphate as selenium donor. The SelA homolog appears to be restricted to these two *Methanothermobacter *species, whereas a CDS for the SelD homolog has been found in every methanogen genome sequenced to date. In nonselenoprotein-containing methanogens, SelD may function in the synthesis of selenouridine-modified tRNAs and/or of selenium-dependent molybdenum hydroxylases, which some methanogens could contain [13, 39, 40].

*Methanothermobacter* species are not only found in anaerobic sewage sludge but also in anoxic freshwater sediments [17]. In these anoxic environments, the temperature is usually below 20°C and thus well below the observed temperature growth range of thermophiles. The origin of *Methanothermobacter *species in such mesophilic and psychrophilic habitats is uncertain. It is possible that the thermophiles originated from thermophilic anoxic environments, such as nonmarine hot springs [42], but when this could have occurred is unknown.

The kinetics and energetics of growth only of *M. marburgensis* have been analyzed in detail. The methanogen grows at 65°C with doubling times of 1.6 h when optimally gassed with 80% H_2_ and 20% CO_2_ at 10^5^ Pa [27]. The apparent *K*_m_ determined with growing cultures is 20% H_2_ and 10% CO_2_ [27]. From the apparent *K*_m_, it can be calculated that the doubling time increases to over 100 days when the H_2_ partial pressure is below 10 Pa, as in the anaerobic sewage sludge from which the organism was first isolated. From the digester dilution rates, a doubling time of at least 30 days is predicted. At 10 Pa, the free energy change associated with reaction 1 is only −40 kJ mol^−1^, which can support the synthesis of less than one ATP from ADP and inorganic phosphate.

## 4. Comparison of the *M. marburgensis* and *M. thermautotrophicus* Genomes

**Figure 1:**
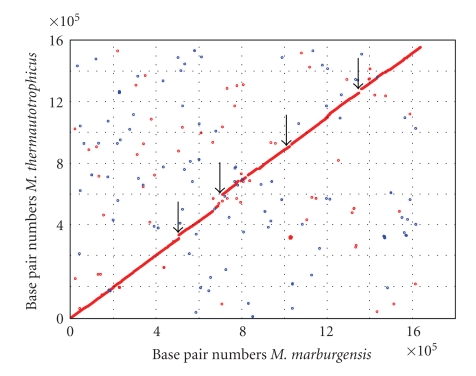
Synteny between the genomes of *Methanothermobacter marburgensis *and* Methanothermobacter thermautotrophicus. x*-axis: position of CDS on the genome of *M. marburgensis; y*-axis: position of homologous CDS on the genome of *M. thermautotrophicus. *The chromosome of *M. thermautotrophicus* (extracted from NC_000916) was rearranged so that it starts at the corresponding CDS encoding the Cdc6 protein. Colinear similarities are depicted by red dots and anti-parallel similarities by blue dots. The synteny plot was produced by the programs of the MUMmer suite [51]. The CDS not in common are dispersed around the two genomes; many are concentrated at the four genome areas indicated by the four arrows.

To compare the genomes of *M. marburgensis *and* M. thermautotrophicus*, we used a two-step approach. In the first step, we used a bidirectional search method that identifies the most similar protein and RNA (tRNA, rRNA, and ribozyme) encoding sequences in the two genomes and allows a sequence to be used only once in the comparison. Each pair of CDS identified in this way was kept. Neither the second- nor third-best hits nor CDS homologs within the same organism were considered. Therefore, if a sequence is not common to the two organisms, this does not mean that there are no paralogs or orthologs of this sequence in the two organisms. Two sequences with a basic local alignment search tool (BLAST) expectation value (*E*-value) in the NCBI database less than 10^−8^ were considered to be of a common origin. A cutoff at lower *E*-values, for example, at an *E*-value of 10^−25^, would have resulted in 40 fewer common CDS, and among these would have been several CDS for proteins with a known function in both organisms, for example, for four ribosomal proteins. In the second step, we aligned full-length sequences using optimal global alignment [43]. Pairs of proteins with full-length alignments with ≥10% identity at the amino acid level were considered as putative orthologous proteins. Using this method and cutoffs, the genomes of *M. marburgensis* and *M. thermautotrophicus* were found to have 1,607 CDS in common, 411 CDS not in common, 39 RNA-coding sequences in common and 1 RNA-coding sequence not in common (Table 1). The two genomes show a high degree of synteny (Figure 1). 

## 5. CDS in Common

Of the 1,607 CDS common to *M. marburgensis* and *M. thermautotrophicus,* some encode proteins with identical or almost identical sequences, and others encode proteins with only a low level of sequence identity, which reflects either large differences in sequence divergence or orthologous gene replacements. Only approximately 57% of the deduced amino acid sequences of the common CDS have *E*-values <10^−100^ and corresponding optimal global similarity-alignment scores >89.2%; approximately 28% have *E*-values between 10^−100^ and 10^−50^ and similarity-alignment scores between 89.2% and 78.3%; approximately 21% have *E*-values between 10^−50^ and 10^−25^ and similarity-alignment scores between 78.3% and 50.0%; 3.7% have *E*-values between 10^−25^ and 10^−8^ (cutoff) and similarity-alignment scores between 50% and 10%; only 3.7% have a similarity-alignment score of 100. These results indicate that many of the proteins in the two organisms have undergone extensive mutations without having lost their function or that these proteins have no essential function and could, therefore, accumulate mutations extensively. Approximately 30% of the CDS in common encode conserved hypothetical proteins.

### 5.1. CDS for Membrane Proteins and Protein Export

Approximately 330 of the 1607 CDS in common are predicted to form at least one transmembrane helix indicating their location in the cytoplasmic membrane. Most of the other CDS appear to encode for cytoplasmic proteins. Only very few CDS appear to have a “periplasmic” location. Both genomes lack CDS for a Tat (twin arginine translocation) system involved in the export of proteins with prosthetic groups such as iron-sulfur centers that can only be assembled in the cytoplasm. The lack of a Tat system appears to be a general property of all methanogens lacking cytochromes. Therefore, the two *Methanothermobacter *species probably do not contain redox-active proteins whose active sites face outwards. This is an issue since there are reports suggesting that one member of the Methanobacteriales, *Methanobacterium palustrae, *can pick up electrons from the surface of electrodes and use these electrons for the reduction of CO_2_ to methane [44, 45]. Interesting in this respect is the finding that *M. marburgensis* and *M. thermautotrophicus* contain a complete Sec protein export system (Table 1). In principle, the methanogens could, therefore, produce electron-conducting fimbriae (nanowires) [46–48] that transfer electrons from the electrode to a cytoplasmic electron acceptor. There is evidence that *M. thermautotrophicus* can form fimbriae with which the organism may attach to H_2_-forming bacteria [49]. Whether these fimbriae can function as nano-wires is not known, and it is also not known whether *M. palustrae *has fimbriae when it picks up electrons from electrode surfaces.

### 5.2. Methanogen-Specific CDS

In the genomes of* M. marburgensis* and *M. thermautotrophicus,* there are 27 CDS and 7 homologous pairs of CDS found in all methanogenic archaea but not in nonmethanogens. These are listed in supplementary  Table 1 (see Table 1 in Supplementary Material available online at doi: 10.1155/2011/973848).

Of the methanogen-specific CDS, many are for proteins involved in CO_2_ reduction with H_2_ to methane. These are the CDS for five of the subunits (MtrA-E) of methyl-H_4_MPT:coenzyme M methyltransferase, the CDS for the three subunits (McrABG and/or MrtABG) of methyl-coenzyme isoenzyme I and II, the CDS for McrC, MrtC, McrD, and/or MrtD and AtwA1 and/or AtwA2 associated with methyl-coenzyme M reductase function, the CDS for FrhB and FrhG of F_420_-reducing hydrogenase and the CDS for ComE of sulfopyruvate decarboxylase involved in coenzyme M biosynthesis. 

The other methanogen-specific CDS are for a predicted molybdenum-iron protein (NflD) homologous to NifD, for a radical-S-adenosylmethionine (SAM) protein homologous to NifB, for a homolog of selenophosphate synthetase (SelD), for a methyltransferase related protein (MtxX), for a peptidyl-prolyl *cis-trans* isomerase-related protein, for a predicted UDP-*N*-acetylmuramyl pentapeptide synthase, for a predicted DNA-binding protein, for a predicted metal-binding transcription factor, for a predicted phosphomannomutase and for 12 conserved hypothetical proteins. 

One of the methanogen-specific CDS, namely, *mcrA*, is used as a specific marker for methanogenic archaea and anaerobic archaea that contain methyl-coenzyme M reductase and oxidize methane [50].

### 5.3. Methanothermobacter- and Methanobacteriales-Specific CDS

Of the CDS in* M. marburgensis, *177 have a counterpart only in *M. thermautotrophicus;* 140 of these are for hypothetical proteins. Ninety-one CDS have a counterpart only in *M. thermautotrophicus, Methanobrevibacter smithii*, *Methanobrevibacter ruminantium,* and *Methanosphaera stadtmanae,* all members of the order of Methanobacteriales. Of these 91 CDS, 67 are for hypothetical proteins. We expect that the *Methanothermobacter*-specific and the Methanobacteriales-specific CDS are for anabolic (biosynthesis) rather than for catabolic (energy metabolism) functions. An exception is MTBMA_c06120, which is one of three CDS predicted to encode coenzyme F_390_ synthetase in both *Methanothermobacter* species. This enzyme catalyzes the conversion of coenzyme F_420_ to a redox-inactive form, which stops methanogenesis from H_2_ and CO_2_ [52].

## 6. CDS Not in Common

The genome of *M. marburgensis* also contains 145 CDS not present in *M. thermautotrophicus* (Supplementary Table  2), and the genome of *M. thermautotrophicus* also contains 266 CDS not present in *M. marburgensis *(Supplementary Table  3). These CDS not in common are dispersed around the two genomes, but many are concentrated at four genome areas (Figure 1). Their origin was traced back to gene-splitting events (frame shifts caused by single-base insertion/deletion; 15%), gene-deletion events (30%), gene-duplication events (30%), and lateral gene-transfer events (24%). (The percent values given are for *M. marburgensis*; for the method of determination, see the supplement.)

Of the CDS not in common and with an annotated function, 18 CDS in *M. thermautotrophicus *and 1 CDS in* M. marburgensis* are predicted to encode Cas proteins, that is, proteins associated with clustered regularly interspaced short palindromic repeats (CRISPR). CRISPR loci encode small RNAs and are, therefore, described in the following subsection. Many of the CDS not in common are predicted to be involved in cell surface sugar biosynthesis (11 CDS in *M. marburgensis* and 23 CDS in* M. thermautotrophicus*). One CDS specific for *M. thermautotrophicus* is for a fimbria protein (MTH60) [49]. This protein shows low sequence similarity to two CDS in each methanogen (MTBMA_c07820 and MTBMA_c07830; MTH382 and MTH383), which are predicted to encode exported proteins. Whether these proteins also form fimbriae is not known. Only the genome of *M. marburgensis* has 2 CDS for a putative transposase of the IS*630* family (MTBMA_c01240 and MTBMA_c01250) and 15 IS-like elements. The two CDS for the transposase are preceded and followed by palindromic sequences [53].

## 7. RNA-Coding Sequences

The genome of *M. marburgensis* harbors 40 tRNA-coding sequences, whereas that of* M. thermautotrophicus* harbors only 39 tRNA-coding sequences. The extra tRNA in *M. marburgensis* is for serine, for which there are 5 tRNAs in *M. marburgensis *and 4 in *M. thermautotrophicus*. The fifth tRNA-Ser coding sequence lies next to that of another tRNAs for serine with the same anticodon. Therefore, the sequence is most likely the result of a gene-duplication event. 

In both methanogens, three of the tRNA-encoding sequences, specifically those for tRNA-Trp, tRNA-Met, and tRNA-Pro, carry an intron. Accordingly, the genomes of the two methanogens also encode a tRNA-splicing endonuclease (MTBMA_c07000; MTH250).

In the genome of *M. marburgensis,* there is only one CRISPR locus with 36 repeats, located after MTBMA_c02230. In the genome of *M. thermautotrophicus,* there are three CRISPR loci with a total number of 175 repeats (http://genoweb1.irisa.fr/Serveur-GPO/outils/repeatsAnalysis/DOMAIN/indexDOMAIN.php). CRISPR loci encode small CRISPR RNAs (crRNAs) that contain a full spacerflanked by partial repeat sequences. Together with genes encoding Cas (CRISPR-associated) proteins (see above), they protect bacteria and archaea from invasion by phage and plasmid DNA through a genetic interference pathway [54–56]. Interestingly, spacer sequences from the CRISPR region of locus 2 from *M. thermautotrophicus* match to nucleotide sequences found in phage ΨM1 of *M. marburgensis* and in phage ΨM100 of *M. wolfeii* [57]; this is in agreement with the observation that *M. thermautotrophicus *is not lysed by these two phages.

## 8. Genes Involved in CH_4_ Formation from CO_2_ and H_2_ and in Energy Conservation

**Figure 2:**
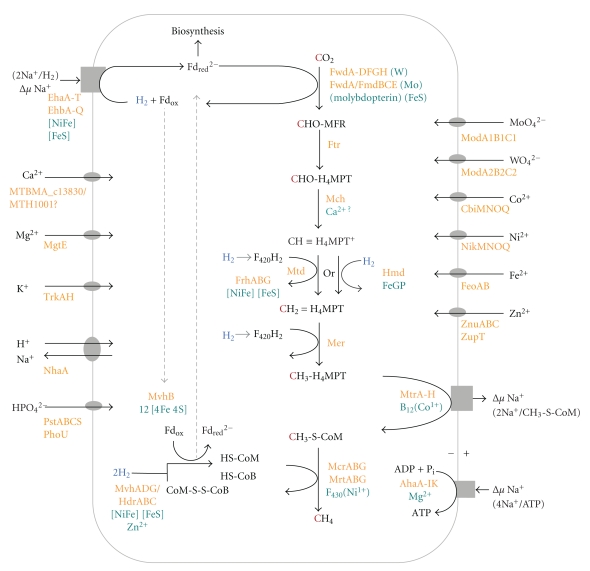
Enzymes, coenzymes, and prosthetic groups involved in the energy metabolism of *Methanothermobacter marburgensis* and *Methanothermobacter thermautotrophicus* during growth on H_2_ and CO_2_. For the synthesis of these components, more than 200 coding sequences are required. A stoichiometry of 4 Na^+^/ATP of the A_1_A_0_-ATP synthase AhaA-IK was assumed to yield 0.5 ATP for every methane generated. This is also predicted from the thermodynamics at physiological H_2_ concentrations. MFR, methanofuran; H_4_MPT, tetrahydromethanopterin; CHO-MFR, formyl-MFR; CHO-H_4_MPT, formy-H_4_MPT; CH *≡* H_4_MPT^+^, methenyl-H_4_MPT^+^; CH_2_ = H_4_MPT, methylene-H_4_MPT; CH_3_-H_4_MPT, methyl-H_4_MPT; Fd, ferredoxin.

Approximately 90 of the annotated CDS present in both *M. marburgensis* and *M. thermautotrophicus*, including those for the methyltransferase MtrA-H, the energy-converting hydrogenases EhaA-T and EhbA-Q, and the A_1_A_0_ ATP synthase AhaA-IK, encode proteins directly involved in CO_2_ reduction to methane with H_2_ and in energy conservation [6]. Another 80 CDS are required for the synthesis of the coenzymes and prosthetic groups, and more than 30 are predicted to have a function in the translocation of ions other than sodium. Their function within energy metabolism is shown in Figure 2. The approximately 200 CDS with an annotated function are listed in Table 2. Other CDS remaining to be identified are also listed in Table 2, with numbers in parentheses, such as those for coenzyme F_430_ biosynthesis and those for posttranslational modifications in the two methyl-coenzyme M reductases.

Some of the approximately 200 CDS also have an anabolic function, such as those for the energy-converting hydrogenases EhaA-T and EhbA-Q (ferredoxin reduction, for example, for CO_2_ reduction to CO) and for the enzymes involved in the reduction of CO_2_ with H_2_ to methyl-tetrahydromethanopterin (methyl-H_4_MPT) (autotrophic CO_2_ fixation). The methyl group of methyl-H_4_MPT is transferred to CO in a coenzyme-A-dependent reaction, yielding acetyl-CoA, from which most cell compounds are synthesized [58].

## 9. Genes for Enzymes Catalyzing H_2_ Activation

In the genomes of *M. marburgensis* and *M. thermautotrophicus,* CDS for five different hydrogenases are found [5] (Figure 2, Table 2). Three are mainly involved in CO_2_ reduction to methane with H_2_ (Figure 2), and two are mainly involved in autotrophic CO_2_ fixation. The three mainly catabolic hydrogenases are (a) the cytoplasmic “methyl-viologen”-reducing [NiFe]-hydrogenase (MvhADG) associated with the heterodisulfide reductase (HdrABC) for the coupled reduction of ferredoxin (Fd) and the heterodisulfide CoM-S-S-CoB with H_2_, (b) the cytoplasmic coenzyme F_420_-reducing [NiFe]-hydrogenase (FrhABG), and (c) the cytoplasmic [Fe]-hydrogenase (Hmd), which substitutes for FrhABG under nickel-limiting growth conditions. The mainly anabolic hydrogenases are the two membrane-associated energy-converting [NiFe]-hydrogenases EhaA-T and EhbA-Q for the reduction of ferredoxin with H_2_.

Under physiological standard conditions [H_2_ partial pressure (pH_2_) = 10^5^ Pa; pH 7], the H^+^/H_2_ couple has a redox potential *E*_0_′ of −414 mV. However, under *in vivo* conditions (pH_2_ *≈*10 Pa; pH 7) (see Section 1), the *E*′ of the H^+^/H_2_ couple is only −300 mV. The *E*_0_′ of the electron acceptors of the hydrogenases are less than −400 mV for ferredoxin (EhaA-T; EhbA-Q), −360 mV for F_420_ (FrhABG), −140 mV for CoM-S-S-CoB (MvhADG/HdrABC), and −380 mV for methenyl-tetrahydromethanopterin (methenyl-H_4_MPT^+^) (Hmd) [6]. Under *in vivo* conditions, the *E*′ of the Fd_ox_/Fd_red_ couple is probably −500 mV, since this is the redox potential of most ferredoxin-dependent reactions in methanogens [6]. Therefore, the reduction of ferredoxin with H_2_ requires energy, that of F_420_ and of methenyl-H_4_MPT^+^ operates near equilibrium [59], and that of CoM-S-S-CoB is exergonic enough to be coupled with energy conservation.

### 9.1. MvhADG

The cytoplasmic [NiFe]-hydrogenase (MvhADG) is frequently referred to as methyl-viologen-reducing [NiFe]-hydrogenase or F_420_-nonreducing hydrogenase. MvhADG is associated with the cytoplasmic heterodisulfide reductase HdrABC, with which the hydrogenase forms a tight complex. The complex catalyzes the CoM-S-S-CoB-dependent reduction of ferredoxin with H_2_ and the ferredoxin-dependent reduction of CoM-S-S-CoB with H_2_. The stoichiometry of ferredoxin (Fd) and CoM-S-S-CoB reduction with H_2_ has been determined to be: 2H_2_ + Fd_ox_ + CoM-S-S-CoB = Fd_red_^2−^ + CoM-SH + CoB-SH + 2H^+^ [60]. Apparently, the MvhADG/HdrABC complex couples the endergonic reduction of ferredoxin with H_2_ to the exergonic reduction of CoM-S-S-CoB with H_2_, and it has been proposed that the coupling proceeds via flavin-based electron bifurcation [6]. The reduced ferredoxin generated in the MvhADG/HdrABC-catalyzed reaction is required for the reduction of CO_2_ to formylmethanofuran (*E*_0_′ = −500 mV) [61]. Evidence was recently provided that MvhADG/HdrABC and formylmethanofuran dehydrogenase form a super complex in the cytoplasm of *Methanococcus maripaludis *[62].

In *M. marburgensis* and in* M. thermautotrophicus,* the genes encoding MvhADG/HdrABC are organized in three nonadjacent transcription units (*mvhDGAB*, *hdrA,* and *hdrBC*). The *mvhDGAB* operon lies directly downstream of the *mtrBDGA* operon, which encodes isoenzyme II of methyl-coenzyme M reductase, and there is evidence that the two operons can be cotranscribed [63, 64].

*mvhA* and *mvhG* encode the large and small hydrogenase subunits, respectively; *mvhD *encodes a [2Fe2S] cluster-containing subunit; and *mvhB* encodes a 12[4Fe4S] polyferredoxin, which is probably the ferredoxin reduced by the MvhADG/HdrABC complex (Figure 2). HdrB harbors the active site for CoM-S-S-CoB reduction and contains zinc and an unusual [4Fe4S] cluster [65]. HdrC harbors two [4Fe4S] clusters, and HdrA contains four [4Fe4S] clusters and FAD. HdrA is considered to be the site of electron bifurcation. Interestingly, HdrA is one of the most highly conserved proteins in all methanogenic archaea and is also found in other archaea and bacteria, which indicates an electron-bifurcating function in these organisms within a different context. Interesting in this respect is that in most methanogens, the CDS for HdrA is located separate from the CDS for HdrBC [66] consistent with HdrA being used not only in combination with HdrBC.

Many members of the Methanomicrobiales lack the genes for the subunits MvhAG. It has been proposed that in these hydrogenotrophic methanogens, FrhAG (see below) rather than MvhAG forms a functional complex with MvhD/HdrABC [5, 67]. The finding that in most methanogens the CDS for MvhADG are located separate from those for HdrA and HdrBC [66] is consistent with HdrABC being used not only in combination with MvhADG for which there is genetic evidence in *Methanococcus maripaludis *[62].

### 9.2. FrhABG

This cytoplasmic [NiFe]-hydrogenase catalyzes the reversible reduction of coenzyme F_420_ with H_2_. The FrhABG complex aggregates to form a complex with a molecular mass of >900 kDa. Upon ultracentrifugation of cell extracts, the F_420_-reducing hydrogenase is recovered in the membrane fraction, which is why it was long believed that this enzyme is membrane associated. In the two *Methanothermobacter* species, the encoding genes are organized in the transcription unit *frhADGB*, where *frhA *encodes the large subunit with the [NiFe] center*, frhG *encodes the small subunit with three [4Fe4S] clusters, and *frhB* encodes an iron-sulfur flavoprotein with one [4Fe4S]-cluster and FAD, which functions as a one electron/two electron switch in F_420_ reduction. The gene *frhD* encodes an endopeptidase (homologous to HycI from *Escherichia coli*), which is required to clip off the C-terminal extension in the FrhA preprotein [68]. The CDS for FrhA and FrhG belong to the methanogen-specific CDS (Supplementary Table  1).

### 9.3. Hmd

The [Fe]-hydrogenase catalyzes the reversible reduction of methenyl-H_4_MPT^+^ with H_2_ to methylene-H_4_MPT. Together with F_420_-dependent methylene-H_4_MPT dehydrogenase (Mtd), the enzyme catalyzes the methenyl-H_4_MPT^+^-dependent reduction of F_420_ with H_2_. The two enzymes can substitute for the F_420_-reducing hydrogenase (FrhABG) under nickel-limiting growth conditions, under which FrhABG is not synthesized [69]. Consistent with this function are the findings that in *M. maripaludis,* it has been possible to knock out the genes for F_420_-reducing hydrogenase, the gene for [Fe]-hydrogenase, or the gene for F_420_-dependent methylene-H_4_MPT dehydrogenase with only minor effects on growth on H_2_ and CO_2_, but it has not been possible to knock out two of these genes [62, 70, 71]. 

Hmd harbors a novel iron-guanylylpyridinol (FeGP) cofactor covalently bound to the homodimeric enzyme only via the thiol/thiolate group of a cysteine residue. The enzyme and the cofactor are not found in most members of the Methanomicrobiales. They appear to be absent in Methanosarcinales and Methanocellales [5].

### 9.4. EhaA-T and EhbA-Q

Both membrane-associated [NiFe]-hydrogenases belong to the group of energy-converting hydrogenases that catalyze the reduction of ferredoxin with H_2_ driven by the proton-motive force or the sodium-ion-motive force [5, 72].* ehaO/ehbN *encodes the large subunit harboring a [NiFe] center, and *ehaN/ehbM* encodes the small subunit characteristic of all [NiFe] hydrogenases. Most of the other* eha* and *ehb *genes encode membrane proteins. In *M. marburgensis, *both enzyme complexes are considered to be sodium-ion dependent and to have a function in providing the cells with reduced ferredoxin mainly for anabolic reactions, such as the reduction of CO_2_ to CO (*E*_0_′ = −520 mV) and of acetyl-CoA plus CO_2_ to pyruvate (*E*_0_′ = −500 mV) [58]. But the two enzymes are probably also required in CO_2_ reduction with H_2_ to methane if or when in the MvhADG/HdrABC-catalyzed reaction less ferredoxin is reduced than required for CO_2_ reduction to formylmethanofuran (*E*_0_′ = −500 mV) (see above). Accordingly, deletion of the *ehb* genes in *M. maripaludis* reveals a function of Ehb in autotrophic CO_2_ fixation: the mutant is an acetate auxotroph. Deletion of the *eha* genes was not possible [73, 74]. In the genomes of *M. marburgensis,* the genes encoding EhaA-T and EhbA-Q are organized in the transcription units *ehaA-T* and *ehbA-Q*, and their transcription is differentially regulated [75].

Four subunits encoded by EhaA-T and EhbA-Q show sequence similarities to the core subunits of the NADH:ubiquinone oxidoreductase (NuoA-N) and the formate hydrogen lyase from *E. coli.* This is why two of these subunits in *M. thermautotrophicus* were annotated in 1997 as NADH dehydrogenase (EhaH/EhbO and EhaJ/EhbF) and two as formate hydrogen lyase (EhaN/EhbM and EhaO/EhbN) [76]. At that time, the subunits EhaS and EhaT were annotated as formylmethanofuran:H_4_MPT formyltransferase and ribokinase, respectively. However, these CDS have been shown to be cotranscribed with the *ehaA*-*R *genes, making a function in CO_2_ reduction to methane (formyltransferase) or sugar activation (ribokinase) very unlikely [75].

Another CDS within the *ehaA*-*T* operon of *M. thermautotrophicus, ehaP2,* encodes a 6[4Fe4S] polyferredoxin that is not found in* M. marburgensis*. In both species, the *ehaA*-*T* transcription unit is followed by a gene for another 6[4Fe4S] polyferredoxin that could be the electron acceptor used by the hydrogenase.

Many members of the Methanomicrobiales lack the genes for EhaA-T and EhbA-Q. Instead these hydrogenotrophic methanogens contain genes for energy-converting hydrogenases different from those found in the other three orders of hydrogenotrophic methanogens [5, 67].

## 10. Genes for Enzymes Catalyzing CO_2_ Reduction to Methane

CO_2_ reduction to methane proceeds via seven steps (Figure 2). Steps one and seven in *M. marburgensis* and *M. thermautotrophicus* are each catalyzed by two enzymes: a tungsten- and a molybdenum-dependent formylmethanofuran dehydrogenase (FwdA-DFGH and FwdA/FmdBCE) and isoenzymes I and II of methyl-coenzyme M reductase (McrABG and MrtABG). The five other steps are each catalyzed by only one enzyme: formylmethanofuran:H_4_MPT formyltransferase (Ftr), methenyl-H_4_MPT^+^ cyclohydrolase (Mch), methylene-H_4_MPT dehydrogenase (Mtd), methylene-H_4_MPT reductase (Mer), and methyl-H_4_MPT:coenzyme M methyltransferase (MtrA-H) (Table 2). The CDS for a second Ftr in *M. thermautotrophicus* [7] turned out to be the subunit EhaT of the energy-converting EhaA-T complex (see above).

### 10.1. FwdA-DFGH and FwdA/FmdBCE

These two cytoplasmic enzymes catalyze the reduction of CO_2_ to formylmethanofuran with reduced ferredoxin. FwdA-DFGH is a tungsten enzyme. FmdBCE is a molybdenum enzyme. In both enzymes, the transition metal is coordinated by two molybdopterin molecules [77]. Interestingly, the tungsten and the molybdenum enzymes share the subunit FwdA, which is synthesized constitutively. In contrast, the molybdenum-dependent enzyme is only synthesized when molybdate is present in the growth medium [78, 79].

### 10.2. Ftr, Mch, Mtd, and Mer

These four cytoplasmic enzymes catalyze the formyl transfer from formylmethanofuran to H_4_MPT (Ftr), the formation of methenyl-H_4_MPT^+^ from formyl-H_4_MPT (Mch), the reduction of methenyl-H_4_MPT^+^ with F_420_H_2_ to methylene-H_4_MPT (Mtd), and the reduction of methylene-H_4_MPT to methyl-H_4_MPT (Mer). They are each composed of only one type of polypeptide and are devoid of a prosthetic group. The cyclohydrolase (Mch) may contain Ca^2+^ [80]. Crystal structures of all four enzymes are available [81].

A CDS (MTBMA_c06530; MTH204) in the genomes of the two *Methanothermobacter* species encodes a putative 5-formyltetrahydrofolate cycloligase present also in the genome of *Methanopyrus kandleri*. The function of the enzyme is unclear, since tetrahydrofolate, a structural and functional analog of H_4_MPT, has not been found in these methanogens [82]. Therefore, the CDS might encode a 5-formyl-H_4_MPT cyclohydrolase with a yet unknown function in reduction of CO_2_ to methane.

### 10.3. MtrA-H

Of the enzymes involved in CO_2_ reduction to methane, only the MtrA-H complex is a membrane enzyme. It is a cobalamin-dependent enzyme with the corrinoid bound to MtrA. The membrane complex couples the exergonic methyl transfer from methyl-H_4_MPT to coenzyme M (Δ*G*°′ = −30 kJ/mol^−1^) with the endergonic translocation of sodium ions [83]. The sodium-ion-motive force thus generated is used to drive the phosphorylation of ADP via the A_1_A_0_ ATP synthase present in all methanogens (see below).

A CDS in the genomes of *M. marburgensis* and *M. thermautotrophicus* encodes the methyltransferase MtxX [84] (MTBMA_c06800 and MTH231), which is also present in the genomes of all other methanogens (Supplementary Table  1). Notably, in some methanogens the *mtxX *gene is in a transcription unit together with *mtxA* and *mtxH*, which are predicted to encode homologs of MtrA and MtrH and are not present in all methanogens. In the MtrA-H complex, MtrH has the function of catalyzing the methyl transfer from methyl-H_4_MPT to the cob(I)alamin bound to MtrA. The function of MtxX remains unknown.

### 10.4. McrABG and MrtABG

The two cytoplasmic nickel enzymes catalyze the reduction of methyl-coenzyme M with coenzyme B. The nickel is bound within factor F_430_, which is the prosthetic group of the two enzymes [4]. Isoenzyme I of methyl-coenzyme M reductase (McrABG) is encoded by the transcription unit *mcrAGCDB,* and isoenzyme II (MrtABG) is encoded by the transcription units *mrtAGDB* (*mrtC* lies outside the transcription unit). The functions of McrC, McrD, MrtC, and MrtD are still unknown [85]. They might be involved in posttranslational modifications, of which there are five within the active-site regions of McrABG and MrtABG (see below). Either one or the other isoenzyme is found in all methanogens and in methanotrophic archaea (Supplementary Table  1).

Methyl-coenzyme M reductases are only active when their prosthetic group F_430_ is in the Ni(I) oxidation state. To render the enzyme from the inactive Ni(II) state to the active Ni(I) state by reduction, several activating enzymes, reduced ferredoxin, and ATP are required. One of the enzymes, component A2 (AtwA), which has an ATP-binding cassette, has been identified [86]. In the genomes of *M. marburgensis* and *M. thermautotrophicus,* two and three CDS, respectively, for AtwA are found (Supplementary Table  1).

## 11. Genes for Electron Transport from H_2_ to Terminal Electron Acceptors

As already indicated, *M. marburgensis* and *M. thermautotrophicus* are devoid of cytochromes and membrane-associated methanophenazine, which would function in electron transport from H_2_ to the electron-accepting steps. The only identified electron carriers are ferredoxins, and several CDS for ferredoxins are found in the genomes of *M. marburgensis* and* M. thermautotrophicus* (Table 2, Figure 2). A 12[4Fe4S] polyferredoxin, which has been characterized, is encoded by *mvhB *of the* mvhDGAB* operon [87]. The transcription units for the energy-converting hydrogenases Eha and Ehb contain CDS for a 6[4Fe4S] polyferredoxin (EhaP) (EhaP twice in *M. thermautotrophicus*), a 10[4Fe4S] polyferredoxin (EhaQ), and a 14[4Fe4S] polyferredoxin (EhbK). The transcription unit for the tungsten-dependent formylmethanofuran dehydrogenase contains a CDS for an 8[4Fe4S] polyferredoxin (FwdF). In the genomes, there are additional monocistronic CDS for an 8[4Fe4S] polyferredoxin, a 6[4Fe4S] polyferredoxin, and four 2[4Fe4S] ferredoxins. The [4Fe4S] clusters within the ferredoxins interact electronically with each other, and different ferredoxins transfer electrons from one to another in spontaneous redox reactions. Electrons from H_2_ can thus probably end up in all of the ferredoxins, from where they, in turn, can be recruited for the reduction of CO_2_ to formylmethanofuran via FwdA/FmdBCE or FwdA-DFGH (Figure 2) and for various anabolic reduction reactions [5].

Anabolic ferredoxin-dependent reactions in *M. marburgensis* and *M. thermautotrophicus *are the reduction of CO_2_ to CO via ferredoxin-dependent CO dehydrogenase (MTBMA_c02870-02930; MTH 1708-1714), pyruvate synthesis from acetyl-CoA and CO_2_ via two pyruvate synthases (MTBMA_c03130-03160 and MTBMA_c09230-09240; MTH1738-1740 and MTH536-537), 2-oxoglutarate synthesis from succinyl-CoA and CO_2_ via 2-oxoglutarate synthase (MTBMA_c14140-14170; MTH1032-1035), synthesis of 2-oxoisovalerate from isobutyryl-CoA and CO_2_ via 2-oxoisovalerate synthase (MTBMA_c10900-10930; MTH703-705), synthesis of indolylpyruvate from indolylacetyl-CoA and CO_2_ via indolylpyruvate synthase (MTBMA_c04220-04230; MTH1852-1853) [32] and N_2_ reduction to NH_3_ via nitrogenase (NifDHK) (MTBMA_c01460, 01490 and 01500; MTH1560, 1563 and 1564). The CDS for these ferredoxin-dependent enzymes are found in the genomes of most but not all methanogens. Thus, for example, acetate-dependent hydrogenotrophic methanogens such as *Methanobrevibacter smithii *and *Methanobrevibacter ruminantium* lack CDS for CO dehydrogenase, and members of the Methanosarcinales lack the CDS for 2-oxoglutarate synthase.

## 12. Genes Involved in Coupling of Methanogenesis with ADP Phosphorylation via the Sodium-Ion-Motive Force

Methanogenesis from CO_2_ and H_2_ is dependent on sodium ions, which are required for coupling methanogenesis with ADP phosphorylation (Figure 2, Table 2). Sodium ions are translocated by four membrane-associated complexes, namely, the methyl-H_4_MPT: coenzyme M methyltransferase complex MtrA-H [83], the energy-converting [NiFe]-hydrogenase complexes EhaA-T and EhbA-Q [75], the A_1_A_0_ ATP synthase complex AhaA-IK, and a sodium ion/proton antiporter NhaA. The methyltransferase appears to translocate two sodium ions per methyl group transferred, as shown by coupling experiments done with vesicle preparations of *Methanosarcina mazei* [83, 88]. The ATP synthase shows a conserved Na^+^-binding motif [89], and it is generally assumed that four sodium ions are required for the phosphorylation of one ADP [90]. Figure 2 shows the proposed reduction of ferredoxin with H_2_ via Eha or Ehb, driven by the sodium-ion-motive force with a Na^+^ to e^−^ stoichiometry of 1; however, this has not yet been established [72]. The sodium/proton antiporter is most likely there for pH homeostasis [91].

## 13. Genes for the Synthesis of Prosthetic Groups of Methanogenic Enzymes

Many of the enzymes catalyzing the reactions involved in CO_2_ reduction to methane with H_2_ contain prosthetic groups that have to be synthesized (Table 2, Figure 2). Prosthetic groups are the [NiFe] centers of [NiFe] hydrogenases (MvhA, FrhA, EhaO, and EhbN), the iron-guanylylpyridinol (FeGP) cofactor in [Fe] hydrogenase (Hmd), the iron-sulfur clusters in [NiFe] hydrogenases, ferredoxins, formylmethanofuran dehydrogenases (Fwd and Fmd), and heterodisulfide reductase (Hdr), molybdopterin in the two formylmethanofuran dehydrogenases (FwdB and FmdB), cobalamin in methyl-H_4_MPT: coenzyme M methyltransferase (MtrA) and F_430_ in methyl-coenzyme M reductase (McrABG and MrtABG). Formyltransferase (Ftr), cyclohydrolase (Mch), methylene-H_4_MPT deydrogenase (Mtd), and methylene-H_4_MPT reductase (Mer) are devoid of a prosthetic group.

### 13.1. [NiFe]-Center

For the synthesis of the [NiFe] center in MvhA, FrhA, EhaO, and EhbN, at least six proteins are required: HypA and HypB for nickel insertion, HypE and HypF for the synthesis of the cyanide ligand from carbamoyl phosphate, and HypC and HypD for the transfer of the cyanide to the active site [68]. The six *hyp* genes are found in all methanogenic archaea; however, they are not clustered as in *E. coli*. In *M. marburgensis* and *M. thermautotrophicus, *only the *hypAB* genes form a transcription unit. 

Both *M. marburgensis* and *M. thermautotrophicus* contain a *carAB* transcription unit. The encoded proteins are probably involved in the synthesis of carbamoyl phosphate from glutamine, bicarbonate, and 2 ATP. A second *carB* gene is probably for the synthesis of carbamoyl phosphate from ammonium, bicarbonate, and 2 ATP. Notably, carbamoyl-phosphate is not only required in methanogens for the synthesis of the active site of [NiFe] hydrogenases but also for the first committed step in pyrimidine and arginine biosynthesis.

### 13.2. FeGP Cofactor

The biosynthesis of the FeGP cofactor (prosthetic group of the [Fe] hydrogenase Hmd) has not yet been elucidated. *In silico* analysis indicates that seven genes co-occurring with the *hmd *gene are involved. In *M. marburgensis *and* M. thermautotrophicus,* six of the *hmd* co-occurring genes (*hcgA-F*) form a transcription unit directly upstream of the* hmd* gene. The gene *hcgG* (MTBMA_c15200; MTH1137) is located five CDS downstream of the *hmd* gene in *M. marburgensis* and four CDS downstream in *M. thermautotrophicus* [5].

The gene *hcgA* (MTBMA_c15270; MTH1143) is predicted to encode a protein with a sequence similar to that of the radical-SAM protein BioB, which is involved in sulfur insertion in biotin biosynthesis [92]. However, HcgA lacks the N-terminal signature motif CX_3_CX_2_C or CX_4_CX_2_C, which is characteristic for the radical-SAM protein superfamily that coordinates a [4Fe4S]-cluster essential for radical formation. Instead, HcgA universally harbors a unique CX_5_CX_2_C motif [92]. The functions of the *hcgB-G* genes remain to be established [5]. The genes encoding Hmd and HcgA*-*G are also found in *Methanobrevibacter smithii*, *Methanobrevibacter ruminantium,* all members of the Methanococcales, *Methanopyrus kandleri,* and one member of the Methanomicrobiales (*Methanocorpusculum labreanum*) [5].

In the genomes of the two *Methanothermobacter* species, two genes homologous to *hmd *are found. The two encoded proteins, HmdII and HmdIII, show only low sequence identity (<20%) to [Fe] hydrogenase but share high sequence identity (80%) with each other. The homologs are not found in methanogens without an *hmd* gene. Structure predictions indicate that HmdII and HmdIII have an intact site for FeGP cofactor binding. Indeed, HmdII binds the FeGP cofactor. However, neither HmdII nor HmdIII catalyze the reduction of methenyl-H_4_MPT^+^ with H_2_. These results were interpreted to indicate that HmdII and HmdIII could be scaffold proteins involved in FeGP-cofactor biosynthesis. However, *Methanocorpusculum labreanum*, *Methanobrevibacter smithii, *and* Methanobrevibacter ruminantium*, all of which can synthesize active [Fe]-hydrogenase, do not contain *hmdII* or *hmdIII* genes, which indicates a nonessential function in active Hmd synthesis [93].

### 13.3. Iron-Sulfur Clusters

*M. marburgensis* and *M. thermautotrophicus* contain many iron-sulfur proteins. Amongst these are the hydrogenases, formylmethanofuran dehydrogenases, heterodisulfide reductase, and the ferredoxins involved in CO_2_ reduction with H_2_ to methane (Figure 2). Accordingly, the iron requirement for growth of the two methanogens is very high [94]. How iron-sulfur clusters are assembled in methanogens is still a mystery. In bacteria, two independent systems, Suf and Isc, have this function [95]. In the two *Methanothermobacter* species, only CDS for a cysteine desulfurase homolog (IscS/SufS), a SufB/SufD homolog (persulfide acceptor), and a SufC homolog (ABC-type ATPase) were found. Additionally, in the genome of *M. thermautotrophicus,* there is a homolog of the bacterial *apbC*/eukaryotic NBP35 gene that encodes an iron-sulfur cluster transfer protein [96, 97]. However, this ApbC homolog is not found in *M. marburgensis* (Table 2). In *Methanococcus maripaludis,* which lacks genes for cysteine desulfurase, cysteine has been shown not to be the sulfur source for the biosynthesis of iron-sulfur clusters and methionine [98].

### 13.4. Molybdopterin

The biosynthesis of molybdopterin appears to proceed as in bacteria, starting from GTP [99]. CDS for MoaABCE and MoeAB (molybdopterin cofactor biosynthesis proteins) and for MobAB (molybdopterin-guanine dinucleotide biosynthesis proteins) are found (Table 2).

### 13.5. B_12_ Cofactor

The corrinoid in *M. marburgensis* was identified to be 5′-hydoxybenzimidazolyl-cobamide (factor III) [100]. It is the prosthetic group of the membrane associated MtrA-H complex (bound to MtrA) and is bound as coenzyme B_12_ to adenosylcobalamin-dependent ribonucleotide reductase (MTBMA_c10320; MTH652), the only protein in *M. marburgensis* and *M. thermautotrophicus* encoded by a gene with an intein sequence (Table 1). Cobalamin biosynthesis starts from glutamate, and most steps appear to follow the anaerobic pathway elucidated in *Salmonella typhimurium,* with *δ*-aminolevulinic acid, uroporphyrinogen-III, and precorrin-2 as intermediates [101–106]. CDS for HemA-D, CysG, CbiA-H, CbiJ, CbiL-Q, CbiT, CbiX, CobN, and CobS are present. The CDS for cobalamin biosynthesis in methanogens are not clustered.

### 13.6. Cofactor F_430_

The synthesis of the nickel tetrapyrrole is predicted to branch off the cobalamin pathway at the intermediate precorrin-2 (dihydrosirohydrochlorin), where also the biosynthesis of siroheme (prosthetic group of assimilatory sulfite reductase) branches off (CysG1 = MTBMA_c06180; MTH167) [107]. Only one of probably six intermediates has been identified [108], and the enzymes involved are not yet known. Two chelatases structurally related to the cobalt chelatase CobNS [109] could be nickel chelatases that catalyze the incorporation of Ni^2+^ into precorrin-2 or a precorrin-2 product (MTBMA_c10550-10570, 09440; MTH673, 556). It has been proposed that proteins structurally related to the nitrogenase [Fe]-protein NifH and the [MoFe]-protein NifDK could have a function in pyrrole ring reduction involved in the synthesis of F_430_ from precorrin-2 [110]. The proposal is based on the finding that homologs of NifD and NifH are involved in protochlorophyllide reduction in phototrophs [111] and that *nifD-* and *nifH*-like genes (*nflD *and *nflH*) (MTBMA_c01050 and 10230; MTH1522 and 643) are present in all methanogens, also in those that lack *nif* genes.

A small number of CDS for conserved hypothetical proteins are found in every genome of methanogenic archaea (Supplementary Table  1) and in the meta-genome of methanotrophic archaea [112] but are not found in any other organism. Among these methanogen-specific CDS, to which also NflD belongs, could be some that function in F_430_ biosynthesis because F_430_ has not been found outside methanogenic archaea and the phylogenetically closely related methanotrophic archaea [113, 114].

## 14. Genes for the Synthesis of Methanogenic Coenzymes

In *M. marburgensis* and *M. thermautotrophicus,* five coenzymes are involved in CO_2_ reduction with H_2_: coenzyme F_420_, methanofuran (MFR), tetrahydromethanopterin (H_4_MPT), coenzyme M (CoM-SH), and coenzyme B (CoB-SH) (Table 2, Figure 2).

### 14.1. Coenzyme F_420_

The biosynthesis of the 5′-deazaflavin derivative starts with a pyrimidine intermediate of riboflavin biosynthesis and the 4-hydroxyphenylpyruvate precursor of tyrosine, yielding after several steps 7,8-didemethyl-8-hydroxy-5-deazariboflavin (F_0_) [115]. F_0_ synthesis involves the radical-SAM proteins CofG and CofH [116]. F_0_ is converted to F_420 _in five enzymatic steps starting from F_0_ and l-lactate [117–120]. The five enzymes involved are CofA-E. The CDS for CofB, which catalyzes the formation of 2-phospho-l-lactate from l-lactate, has not yet been identified. In *M. marburgensis* and *M*. *thermautotrophicus,* a CDS for CofF that is present in other methanogens is not found. CofF encodes a *γ*-F_420_-2:*α*-l-glutamate ligase and caps the *γ*-glutamyl tail of coenzyme F_420_ [121]. This is not required in the Methanobacteriales, in which F_420_ is not capped [4]. Associated with F_420_ function are CDS predicted to encode three coenzyme F_390_ synthetase isoenzymes (MTBMA_c01110, 04250, 06120; MTH161, 1528, 1855). F_390_ synthase catalyzes the conversion of coenzyme F_420_ to a redox-inactive form, which stops methanogenesis from H_2_ and CO_2_ [52].

In *M. marburgensis* and *M. thermautotrophicus,* coenzyme F_420_ not only functions in CO_2_ reduction to methane with H_2_ (Figure 2) but also in O_2_ detoxification [122] and in biosynthesis [123]. Both methanogens contain CDS for the three F_420_H_2_ oxidases FprA1–3 (MTBMA_c06080, 06690, 17400; MTH157, 220, 1350) [124], F_420_-dependent sulfite reductase Fno (MTBMA_c07290; MTH280) [125], F_420_-dependent glutamate synthase (MTBMA_c06440; MTH193) [126] (remains to be proven by purification and sequencing), and F_420_-dependent formate dehydrogenase FdhAB (MTBMA_c15220 and 15230; MTH1139 and 1140) [127]. The latter three enzymes show domains with sequence similarity to the subunit FrhB of F_420_-reducing hydrogenase, which carries the binding site for the prosthetic groups F_420_, FAD, and a [4Fe4S] cluster. Therefore, sulfite reductase and the subunit 4 of F_420_-dependent glutamate synthase and sometimes also subunit B of formate dehydrogenase have previously been annotated as FrhB.

The two *Methanothermobacter* species have CDS for formate dehydrogenase even though they cannot grow on formate. They require the enzyme for CO_2_ reduction to formate, which in turn is required for the synthesis of purines and as an electron donor for anaerobic ribonucleotide reductase (class III) [82]. Methanogens without cytochromes incorporate formate into C^2^ of purines in an ATP-dependent reaction with formyl phosphate as intermediate, as catalyzed by 5-formaminoimidazole-4-carboxamide-1 *β*-d-ribofuranosyl 5′-monophosphate synthetase (PurP) (MTBMA_c15790; MTH1201) [128]. CO_2_ reduction to formate is their only means of generating formate. Accordingly, formate-dehydrogenase-negative mutants of *M. marburgensis* require formate for growth on H_2_ and CO_2_ [28]. *M. marburgensis* and *M. thermautotrophicus* contain CDS for a second formate dehydrogenase subunit FdhA and a formate dehydrogenase accessory protein FdhD, but they lack a CDS for a formate carrier (FdhC) [129, 130], which is present in methanogens that can grow on formate.

### 14.2. Methanofuran

The pathway for the biosynthesis of methanofuran and the responsible genes have yet to be identified. A clear structural element in all known methanofurans is tyramine, likely produced by the decarboxylation of l-tyrosine [131]. In *M. marburgensis *an*d M. thermoautotrophicus, *the decarboxylation is catalyzed by MfnA.

### 14.3. Tetrahydromethanopterin

7,8-Dihydro-d-neopterin 2′,3′-cyclic phosphate is the first intermediate in the biosynthesis of the pterin portion of tetrahydromethanopterin. This intermediate is produced from GTP by MptA, a new class of GTP cyclohydrolase I, and is further hydrolyzed via the cyclic phosphodiesterase MptB to a mixture of 7,8-dihydro-d-neopterin 2′-monophosphate and 7,8-dihydro-d-neopterin 3′-monophosphate [132]. The biosynthesis of the nonpterin portion involves at least nine steps, the first being catalyzed by ribofuranosylaminobenzene 5′-phosphate synthase [133].

### 14.4. Coenzyme M

All but one (ComF) of the CDS required for coenzyme M synthesis (ComA-F) have been identified in the genomes of *M. marburgensis* and *M. thermautotrophicus*. Biosynthesis starts from phosphoenol pyruvate with sulfolactic acid, sulfopyruvic acid, and sulfacetaldehyde as intermediates. ComA catalyzes the Michael addition of sulfite to phosphoenolpyruvate. ComB is a Mg^2+^-dependent acid phosphatase specific for 2-hydroxycarboxylic acid monophosphate esters. ComC catalyzes the oxidation of the (*R*)-sulfolactate intermediate to form sulfopyruvate, which is decarboxylated to produce sulfoacetaldehyde via ComDE. The CDS for ComE is one of the methanogen-specific genes (Supplementary Table  1). The final postulated enzyme in CoM biosynthesis, ComF, which has not yet been identified in any organism, catalyzes the reductive thiolation of sulfoacetaldehyde to coenzyme M, a reaction which most likely does not proceed spontaneously. The absence of *comA, comB,* and *comC* in the genomes of *Methanosarcina *spp. and members of the Methanomicrobiales implies that these methanogens synthesize sulfopyruvate by a different route [116].

### 14.5. Coenzyme B

The biosynthesis of coenzyme B starts from acetyl-CoA and 2-oxoglutarate and proceeds via 2-oxoadipate, 2-oxopimelate, 2-oxosuberate, and suberate semialdehyde as intermediates. CDS for homologs of (*R*)-citrate synthase [134], aconitase, and isocitrate dehydrogenase have been found. There is only one synthase for the three synthase reactions, one isomerase for the three isomerization reactions, and one dehydrogenase for the three dehydrogenation reactions. The three enzymes are homologs of isopropylmalate synthase (LeuA), isopropylmalate isomerase (LeuC/D), and isopropylmalate dehydrogenase (LeuB), respectively, [116, 135] for which there are two annotated gene copies in the genomes of *M. marburgensis* and of* M. thermautotrophicus.*

## 15. Genes for Transport of Ions Required for Growth

The growth of methanogens is dependent not only on sodium ions (see above), but also on nickel, cobalt, iron, magnesium, and potassium cations and on molybdate or tungstate and phosphate anions [26, 27]. Growth is probably also dependent on zinc and calcium cations present as trace contaminations in the growth media. All these ions, all of which are required for the synthesis of enzymes, prosthetic groups, and coenzymes, must be taken up from the growth medium (Table 2, Figure 2).

With respect to Fe^2+^, Co^2+^, Ni^2+^, and Zn^2+^ uptake, it has to be considered that *M. marburgensis* and *M. thermautotrophicus* thrive in habitats, where the H_2_S/HS^−^ concentrations are generally high and the pH is near 7. The transition metal ions in such habitats are mostly present as sulfides, and therefore, the concentrations of the free ions are very low (<10^−8^ M), with the lowest being that of free zinc ions. The solubility product constants are 4.5 × 10^−24^ for ZnS, 2 × 10^−21^ for NiS, 4 × 10^−21^ for CoS, and 6 × 10^−16^ for FeS [136].

### 15.1. Nickel

Nickel ions have to be taken up by the cells for the synthesis of the four different [NiFe] hydrogenases (EhaA-T, EhbA-Q, FrhABG, and MvhADG), the two methyl-coenzyme M reductase isoenzymes (McrABG and MrtABG), and the carbon monoxide-acetyl-CoA synthase/decarbonylase complex (MTBMA_c02870-02930; MTH1708-1714) involved in autotrophic CO_2_ fixation. The ABC transporter involved has not yet been identified. There appears to be no close homolog to the NikA–E nickel transport system in *E. coli*. In the genome of the two *Methanothermobacter *species, there are two sets of CDS (Cbi1 and Cbi2) predicted to encode a Co^2+^ ABC transporter (see below), one of which (CbiM1N1O1Q1) has been proposed to be a Ni^2+^ ABC transporter [102, 137]. But the Ni^2+^ transporter could also be encoded by one of the five sets of CDS for ABC transport systems without an annotated function present in the genomes of *M. marburgensis *(MTBMA_c00690 + 00700, MTBMA_c10830 + 10840, MTBMA_c14760 + 14770, MTBMA_c15330 + 15340, MTBMA_c17570 + 17580) and *M. thermautotrophicus* (MTH1486 + 1487, MTH695 + 696, MTH1093 + 1094, MTH1149 + 1150, MTH1370 + 1371).

### 15.2. Cobalt

Cobalt ions are required for the synthesis of cobalamin in the MtrA-H complex and of coenzyme B_12_ in the adenosyl cobalamin-dependent ribonucleotide reductase. They are most probably taken up by the ABC transporter CbiMNOQ [137].

### 15.3. Iron

Ferrous ions for the synthesis of iron-sulfur clusters in the [NiFe] hydrogenases, formylmethanofuran dehydrogenases, heterodisulfide reductase, ferredoxins, and [Fe] hydrogenase are thought to be taken up by the ATP-driven FeoAB transport system encoded by *feoAB* [138].

### 15.4. Zinc

Of the proteins involved in CO_2_ reduction with H_2_ to methane only the subunit B of heterodisulfide reductase contains zinc [65]. But zinc ions are also required for RNA polymerase and other biosynthetic enzymes. The gene cluster for the putative high-affinity Zn^2+^ ABC transporter ZnuABC/ZupT in *M. marburgensis* and *M. thermautotrophicus* lies next to an open reading frame for the nickel-responsive transcriptional regulator NikR homolog (MTBMA_c09830; MTH603). Therefore, the NikR homolog might in reality be a zinc-responsive regulator [139]. NikR from *E. coli* also binds zinc ions, but without a conformational change response [140].

### 15.5. Magnesium

Magnesium ions are required in ATP- and ADP-dependent reactions, because synthetases and kinases generally use complexes of ATP and ADP with Mg^2+^ as substrates and products. Mg^2+^ is predicted to be taken up by the MgtE system [141].

### 15.6. Calcium

The crystal structure of Mch from *Methanopyrus kandleri *revealed the presence of a structural calcium ion [80]. Methane formation in cell suspensions of *M. thermautotrophicus* is stimulated by Ca^2+^ [142]. These findings indicate a function of Ca^2+^ in methanogenesis. A membrane-associated Ca^2+^ ATPase has been identified via bioinformatic methods [143]. Available evidence indicates that Ca^2+^ uptake is inhibited by Ni^2+^ and Co^2+^ [142]. If a Ca^2+^ uptake system is present, it must be a high-affinity uptake system, since media for the growth of *M. marburgensis* do not have to be supplemented with calcium salts for the methanogen to grow optimally [27]. The contaminating calcium ion concentration in the media has been determined to be 0.5 *μ*M [142].

### 15.7. Potassium

Potassium ions are not directly involved in methanogenesis from CO_2_ and H_2_O, but most of the methanogenic enzymes function optimally only at high K^+^ concentrations. In growing *M. marburgensis* cells, the intracellular K^+^ concentrations have been determined to be above 0.5 M [144]. The potassium ions are most probably taken up by the low-affinity TrkAH system [145], for which CDS in the genomes of the two *Methanothermobacter *species have been found.

### 15.8. Molybdate and Tungstate

Molybdate ions are required for the synthesis of the molybdenum-dependent formylmethanofuran dehydrogenase, formate dehydrogenase, and nitrogenase. MoO_4_^2−^ is most likely taken up by the ABC transporter ModA1B1C1 [146, 147] encoded by the CDS located directly adjacent to the CDS for molybdenum-dependent formylmethanofuran dehydrogenase. Tungstate ions are required for the synthesis of the tungsten-dependent formylmethanofuran dehydrogenase. WO_4_^2−^ is most likely taken up by the ABC transporter ModA2B2C2 [148, 149].

### 15.9. Phosphate

In methanogenesis from CO_2_ and H_2_, phosphate is required in ATP formation via the A_1_A_0_ ATP synthase and for the synthesis of the coenzymes H_4_MPT, coenzyme B, and the FeGP-cofactor, which contain covalently bound phosphate. The phosphate is probably taken up by a PstABCS/PhoU system [150].

## 16. Transcriptional Regulation and Posttranslational Modifications

Up to here, the regulation and posttranslational modifications of enzymes involved in CO_2_ reduction with H_2_ to methane have only been mentioned. They are, in the following, dealt with in more detail.

### 16.1. Transcriptional Regulation

Mainly, the effects of changes of the Ni^2+^ and molybdate concentration and in the H_2_ partial pressure on transcription during growth of *M. marburgensis* and *M. thermautotrophicus* have been studied.

#### 16.1.1. Regulation by Ni^2+^

Transcription of the genes for F_420_-dependent methylene-H_4_MPT dehydrogenase (Mtd), F_420_-reducing hydrogenase (FrhABG), and H_2_-forming methylene-H_4_MPT dehydrogenase (Hmd) is regulated by Ni^2+^ [151]. When *M. marburgensis* grows under nickel-limiting conditions, transcription of the *mtd *and* hmd *genes is upregulated and that of the *frhADBG *genes is downregulated [151]. The genome of *M. marburgensis* has three CDS and the genome of *M. thermautotrophicus* has two CDS predicted to encode a nickel-responsive transcriptional regulator (NikR) [139]. In bacteria, NikR regulates transcription of genes involved in the synthesis of nickel enzymes and nickel transport [152]. The putative presence of several nickel responsive regulators might reflect the methanogens' use of an unusually high number of different nickel proteins and their growth in habitats where the nickel concentration is sometimes growth limiting [5].

#### 16.1.2. Regulation by Molybdate

Transcription of the *fmdBCE* genes of the molybdenum-dependent formylmethanofuran dehydrogenase (FwdA/FmdBCE) responds to the availability of molybdate in the growth medium. The *fmdECB* operon in *M. marburgensis* and *M. thermautotrophicus* is directly preceded by the open reading frame *tfx*, predicted to encode a DNA-binding protein. Tfx binds specifically to nucleotide sequences downstream of the promoter of the *fmdECB *operon. Northern blot hybridizations have revealed that transcription of *tfx *is repressed in the presence of tungstate [153].

#### 16.1.3. Regulation by H_2_

Transcription of the *frhADBG *genes has been shown to be upregulated under H_2_-limiting conditions [151]. Also, the transcription of the genes for the synthesis of the two methyl-coenzyme M reductase isoenzymes McrABG and MrtABG responds differentially to the availability of H_2_. MrtABG is preferably synthesized in the early exponential growth phase and McrABG preferably in the late exponential growth phase [154, 155]. A plausible candidate for a transcriptional regulator of the *mcr *operon in *M. thermautotrophicus* was recently shown to be the inosine-monophosphate dehydrogenase related-protein IMPDH VII encoded by MTH126 (= MTBMA_c05760). IMPDH VII, which binds to the promoter region of the Mcr-encoding operon, is predicted to have a winged helix-turn-helix DNA-binding motif and two cystathionine *β*-synthase (CBS) domains, and has been suspected to be an energy-sensing module [156]. A sensor for H_2_ has not been found. The genomes of *M. marburgensis* and *M. thermautotrophicus* lack CDS for a sensory hydrogenase as found in *Ralstonia eutropha *[157].

#### 16.1.4. Regulation by Nitrogen

Of the many transcriptionally regulated anabolic genes, only those involved in nitrogen assimilation are addressed here because they are relevant for the growth properties of *M. marburgensis* and *M. thermautotrophicus*. Within the CDS cluster for the nitrogenase function (MTBMA_c01460-01530; MTH1560-1566), two CDS for nitrogen regulation (NifI_1_ and NifI_2_) are present (MTBMA_c01470-01480; MTH1561-1562) that regulate expression of genes involved in N_2_ fixation and NH_3_ assimilation [158]. Upstream of the cluster, a CDS for the global nitrogen repressor NrpR is found (MTBMA_c01560; MTH1569) [159]. NrpR is predicted to bind to the inverted repeat operators of the nitrogenase structural genes *nifHDK* (MTBMA_c01460, 01490, 01500; MTH1560, 1563, 1564), the glutamine synthetase gene *glnA* (MTBMA_c01570; MTH1570), and the ammonium transporter genes *amt1 *(MTBMA_c10420; MTH661) and *amt2* (MTBMA_c10450; MTH663). Directly upstream of each of the two *amt* genes lies a CDS for one of two transcriptional regulators, GlnK1 and GlnK2. This indicates that in the two *Methanothermobacter* species, nitrogen assimilation is tightly regulated. This could explain why attempts to grow *M. marburgensis* on N_2_ as sole nitrogen source have failed (unpublished results).

### 16.2. Posttranslational Modifications

Of the proteins involved in CO_2_ reduction with H_2_ to methane the four [NiFe] hydrogenases and the two methyl-coenzyme M reductases have been found to be posttranslationally modified.

#### 16.2.1. Hydrogenases

In the two *Methanothermobacter* species, the large subunit of all three subtypes of [NiFe] hydrogenases (EhaO, EhbN, MvhA, and FrhA) (Table 2) is synthesized as a preprotein, from which a C-terminal sequence has to be clipped off after the DPCxxCxxH/R motif involved in [NiFe] center coordination. This is the last step in [NiFe] center synthesis [68]. Therefore, genes for four hydrogenase-specific endopeptidases should be present. In *M. marburgensis* and *M. thermautotrophicus,* only the endopeptidase encoded by *frhD* (MTBMA_c16850; MTH1299) in the *frhADGB *transcription unit could be unambiguously identified. In addition to *frhD* in the *frhADGB* operon, a second *frhD* gene (MTBMA_c11320; MTH737) (homologous to* hycI *in *E. coli*) and several other genes for metalloproteases outside the transcription units for the four [NiFe] hydrogenases are found.

#### 16.2.2. Methyl-Coenzyme M Reductases

In the structure of the two methyl-coenzyme M reductase isoenzymes (Mcr and Mrt), a thioglycine, a C^2^-methyl alanine, a C^5^-methyl arginine, an *N*-methyl histidine, and an *S*-methyl cysteine are found in the *α*-chain [160]. The methyl groups are posttranslationally introduced from *S*-adenosylmethionine (SAM) [161, 162]. The formation of C^2^-methyl alanine and C^5^-methyl arginine involves a C-methylation and is, therefore, predicted to involve radical SAM enzymes [163]. The formation of *N*-methyl histidine and *S*-methyl cysteine is predicted to involve SAM dependent methyltransferases. The genomes of *M. marburgensis* and *M. thermautotrophicus* encode at least 14 radical-SAM enzymes and more than 15 SAM-dependent methyltransferases; a function for most of these has not yet been assigned. SAM is synthesized by an archaeal-type SAM synthetase [164].

## 17. Conclusions

We identified approximately 200 CDS in *M. marburgensis* and *M. thermautotrophicus *that encode proteins directly or indirectly involved in CO_2_ reduction to methane with H_2_ and in coupling this process with energy conservation (Figure 2). More than 50 of these CDS are concentrated in the genome region between MTBMA_c16880 and MTBMA_c15110 and between MTH1302 and MTH1128, whereas the others are scattered all over the genome. Approximately 90 CDS are for membrane-associated protein complexes, of which only the MtrA-H complex has been purified. Crystal structures of many of the cytoplasmic enzymes catalyzing CO_2_ reduction to methane have been determined [81, 93, 123]. However, of the biosynthetic proteins involved in coenzyme and prosthetic group synthesis, only a few have been characterized, and approximately 20 have not yet even been identified. The lack of a genetic system for the two *Methanothermobacter *species presently allows their identification only by reverse genetics.

The comparison of the genomes of two *Methanothermobacter *species has revealed that they are pretty much the same in all core catabolic and anabolic reactions. Some of the 1,607 CDS in common encode proteins with identical or almost identical sequence; others, however, encode proteins with only low sequence identity. The differences in phenotype observed, such as differences in growth rate and in ATPase activity, can, therefore, easily be the result of these sequence differences, since even point (single nucleotide) mutations can result in a change in the phenotype. But it is also likely that some of the differences, such as in cell wall sugar composition and susceptibility to phage infection, lie hidden within sequences that the two organisms do not have in common. Of these, those for the synthesis of cell surface polysaccharides and for IS-like elements and CRISPR are most apparent.

Our comparison of the genomes provides a roadmap for defining the majority of functional components responsible for the methanogenic phenotype in *M. marburgensis *and *M. thermautotrophicus *and a template for metabolic pathway reconstruction and gene discovery in comparisons of clonal populations or meta-genomes. Most of the 200 genes that have a direct or indirect function in CO_2_ reduction with H_2_ to methane are also found in the Methanococcales and Methanopyrales, whereas some are lacking in the Methanomicrobiales. Thus, many members of the Methanomicrobiales lack the genes for the heterodisulfide-reductase (HdrABC-) associated hydrogenase subunits MvhAG and many contain genes for energy-converting hydrogenases different from those found in the other three orders of hydrogenotrophic methanogens [5, 67]. Interestingly, the cytochrome-containing Methanocellales appear to be more similar with respect to their core catabolic genes to the Methanobacteriales than to the cytochrome-containing Methanosarcinales.

It was first thought that most of the reactions, coenzymes, and prosthetic groups involved in CO_2_ reduction with H_2_ to CH_4_ in *M. thermautotrophicus *and *M. marburgensis *would be unique to methanogens. Only much later was it discovered that “methanogenic” genes are also present in other archaea and in bacteria. For example, the sulfate-reducing *Archaeoglobus fulgidus* uses many enzymes and coenzymes in anaerobic lactic acid oxidation to 3 CO_2_ that are also used by methanogenic archaea in CO_2_ reduction to methane [165], and *Desulfobacterium autotrophicum* contains gene clusters for the heterodisulfide reductase HdrABC [166]. Another example is the methylotrophic bacteria, which use methanogenic enzymes and coenzymes in their energy metabolism [167]. Finally, [NiFe] hydrogenases, which were discovered first in *M. marburgensis* [168], are also found, for example, in *E. coli* [68, 169]. *M. marburgensis* and *M. thermautotrophicus* are therefore not only model organisms for the study of methanogenesis from H_2_ and CO_2_ but also for the study of H_2_ and C_1_ metabolism in general.

## Supplementary Material

The methods of sequencing employed and the data basis used in the sequence comparisons are described. A list of methanogen specific genes is given (supplementary Table 1). It is described how the CDS not in common were traced back to putative gene splitting events, gene deletion events, gene duplication events and lateral gene transfer events. For *Methanothermobacter marburgensis*, CDS not in common are listed in supplementary Table 2 and those for *M. thermoautotrophicus* in supplementary Table 3.

## Figures and Tables

**Table 1:** Genomes of *Methanothermobacter marburgensis* and of *Methanothermobacter thermautotrophicus. *

	*M. marburgensis*	*M. thermautotrophicus*
Chromosome size (bp)	1, 639,135	1,751,377
G+C content (mol%)	48.64	49.54
Coding (%)	90.94	91.02
CDS	1,752	1,873
CDS in common	1,607	1,607
for conserved hypothetical proteins	468	468
for predicted membrane proteins^a^	327	327
CDS not in common	145	266
for predicted membrane proteins^a^	48	80
CDS for proteins with an intein	1^b^	1^b^
5S rRNA	3	3
16S rRNA	2	2
23S rRNA	2	2
tRNA	40	39
tRNA with introns	3	3
Catalytic RNA (Ribonuclease P)	1	1
Rnp-assisting proteins	4	4
CRISPR locus	1 (36 repeats)	3 (175 repeats)
CRISPR-associated CDS	1	18
Sec transport system	yes	yes
7S rRNA (signal recognition particle RNA)	1	1
SRP-19, SRP-54, five Sec proteins	yes	yes
FtsY (SRP receptor); YidC	yes	yes
Signal peptidase	1	1
Tat system	no	no
Fimbrin	2	3
Sortase	2	2
Adhesin-like proteins^c^	12	12
IS-like elements	15	no
Transposase	1 (2 CDS)	no
Plasmid	pME2001 (4,439 bp)	no

^a^CDS predicted to code for transmembrane proteins based on the presence of at least one transmembrane helix as determined by TMHMM Server v. 2.0. ^b^adenosylcobalamin-dependent ribonucleotide reductase (MTBMA_c10320; MTH652).^c^homologous to one or several of the 105 adhesin-like proteins in *Methanobrevibacter ruminantium* [41].

**Table 2:** The ca. 200 identified genes required for methane formation and energy conservation in *Methanothermobacter marburgensis* and *Methanothermobacter thermautotrophicus*. The number of CDS that remain to be identified are given in parentheses. For functions, see Figure 2. H_4_MPT, tetrahydromethanopterin; MFR, methanofuran.

Proteins		Genes in *M. marburgensis *		Genes in* M. thermautotrophicus *	
		(MTBMA_c)	Number of CDS	(MTH)	
H_2_ * activation *					

MvhADG: F_420_-non reducing [NiFe]-hydrogenase associated with		15190–15160 (*mvhDGAB)* (MvhB is a polyferredoxin)	4	1136–1133	
HdrABC: heterodisulfide reductase		17680, 04500, 04490	3	1381, 1879, 1878	
FrhABG: F_420_-reducing [NiFe]-hydrogenase; synthesis regulated by nickel		16860–16830 (*frhADGB*) (FrhD is an endopeptidase)	5	1300–1297	
Hmd: [Fe]-hydrogenase (H_2_-forming methylene-H_4_MPT dehydrogenase); synthesis regulated by nickel		15260	1	1142	
EhaA-T: Energy-converting [NiFe]-hydrogenase; membrane-associated; Na^+^-dependent; mainly anabolic function		07840–08030	20	384–404 (EhaP twice)	
EhbA-Q: Energy-converting [NiFe]-hydrogenase; membrane-associated; Na^+^-dependent; mainly anabolic function		16230–16390	17	1251–1235	
Nickel-responsive transcriptional regulator NikR		07330, 09830, 11340	3	603, 739	

CO_2_ * reduction to methane *					

FwdA-DFGH: Tungsten-dependent formyl-MFR dehydrogenase; formed constitutively		01390–01450 (*fwdHFGDACB*)	7	1553–1559	
FwdA/FmdBCE: Molybdenum-dependent formyl-MFR dehydrogenase; formed only in the presence of molybdate, involving DNA-binding protein Tfx		13050–13070 (*fmdECB*)	3	917–919	
		13040	1	916	
Ftr: Formylmethanofuran: H_4_MPT formyltransferase		16460	1	403	
Mch: Methenyl-H_4_MPT cyclohydrolase		11690	1	773	
Mtd: F_420_-dependent methylene-H_4_MPT dehydrogenase; synthesis regulated by nickel		00500	1	1464	
Mer: F_420_-dependent methylene-H_4_MPT reductase		03270	1	1752	
MtrA-H: Methyl-H_4_MPT:coenzyme M methyltransferase*; *membrane-associated; Na^+^-dependent		15400–15470 (*mtrHGFABCDE*)	7	1156–1163	
McrABG: Methyl-coenzyme M reductase isoenzyme I; contains 5 posttranslational modifications		15480–15520 (*mcrAGCDB*)	5	1164–1168	
		(McrCD of unknown function)	(5)		
MrtABG: Methyl-coenzyme M reductase isoenzyme II; contains 5 posttranslational modifications		15120–15150 (*mrtAGDB*)	4	1129–1132	
		(MrtD of unknown function)			
AtwA: Mcr/Mrt-activating enzyme A2		13970, 06010	2	151, 454, 1015	
Putative transcriptional regulator of the *mcr* operon		05760	1	126	

*Electron transport*					

6[4Fe4S] polyferredoxin (EhaP);		See above		See above	
8[4Fe4S] polyferredoxin (FwdF);					
10[4Fe4S] polyferredoxin (EhaQ);					
12[4Fe4S] polyferredoxin (MvhB);					
14[4Fe4S] polyferredoxin (EhbK)					
2[4Fe4S] ferredoxins		00530, 03900, 07270, 14890	4	1468, 1819, 278, 1106	
6[4Fe4S] polyferredoxin		08040	1	405	
8[4Fe4S] polyferredoxin		17360	1	1345	

*ADP phosphorylation via the *Na*^+^-motive force* (generated in the MtrA-H-catalyzed reaction; see above)					

AhaA-IK: A_1_A_0_ ATP synthase complex; membrane-associated; Na^+^-dependent?		13380–13470 (*ahaGDBAFCEKIH*)	10	952–961	
NhaA: Sodium ion/proton antiporter; pH regulation		11540	1	760	

*Synthesis of prosthetic groups of methanogenic enzymes*					

* [NiFe] center of the [NiFe]-hydrogenases*			9		
CarAB: Carbamoyl-phosphate synthase		13790–13800		996–998 (996+997 = CarB)	
HypA-F: Hydrogenase maturation factors		11790, 11780, 02320, 14600,06550 or 01080, 16720		783, 782, 1649, 1072, 205 or 1525, 1287	
FrhD and HycI: endopeptidases		See above and 11320		See above and 737	
*FeGP cofactor of Hmd*			8		
HcgA-G: Hmd co-occurring proteins		15270–15320, 15200 (*hcgABCFED,G*)		1143–1148, 1137	
HmdII and HmdIII		00970, 08950		1512, 504	
*FeS-centers of hydrogenases, Fmd, Fwd, Hdr and ferredoxins*			3		
IscS/SufS: Cysteine desulfurase		17750		1389	
SufB/D and SufC		15340, 15330		1150, 1149	
ApbC: Putative iron-sulfur cluster transfer protein		Not found		1176	
*Molybdopterin cofactor of formyl-MFR dehydrogenase *			10		
MoaABCE		01360, 04310, 12050, 05990		1550, 1861, 809, 149	
MoeAB		13850 or 17560, 01580		1003 or 1369, 1571	
MobAB		05930 or 09160, 01370		143 or 528, 1551	
*B_12_ cofactor of methyltransferase MtrA-H*			4		
HemA: Glutamyl-tRNA reductase		13940		1012	
HemB: *δ*-Aminolevulinic acid dehydratase		11390		744	
HemC: Porphobilinogen deaminase		12690		874	
HemD: Uroporphyrinogen-III synthase		06170		166	
CysG1: Uroporphyrin-III C-methyltransferase (precorrin-2 synthesis)		06180	1	167	
CbiX: Sirohydrochlorin cobalt chelatase		17830	14	1397	
CbiL: Precorrin-2 C(20)-methyltransferase		17380		1348	
CbiH: Precorrin-3B C17 methylase		17900		1403	
CbiG: Cobalamin biosynthesis protein		17950		1408	
CbiF: Precorrin-4 methylase		09820		602	
CbiD: Precorrin-6A synthase		12040		808	
CbiE: Precorrin-6Y methylase		00990		1514	
CbiJ: Precorrin-6X reductase		13840		1002	
CbiT: Cobalt-precorrin-6Y C(15)-methyltransferase		05960		146	
CbiC: Precorrin-8X methylmutase		06760		227	
CbiA: Cobyrinic acid a,c-diamide synthase		00460		1460	
CobS: Cobalamin-5-phosphate synthase		14960		1112	
CbiB: Cobalamin biosynthesis protein		17960		1409	
CobN: Cobalamin biosynthesis protein		06860 or 09040 or 17530		237 or 514 or 1363	
*F_430_ cofactor of methyl-coenzyme reductases* Biosynthesis starts from uroporphyrinogen-III, probably 7 reactions are involved		?	(7)	?	

*Coenzyme biosynthesis*					

*Coenzyme F_420_*			6		
CofA: Lactaldehyde dehydrogenase		13630		978	
CofB: l-Lactate kinase		?	(1)	?	
CofC: 2-phospho-l-lactate guanylyltransferase		09930		613	
CofD: LPPG:F_O_ 2-phospho-l-lactate transferase		14000		1018	
CofE: F_420_-0: *γ*-glutamyl ligase		14010		1019	
CofGH: F_O_ synthase		15760,12170		1198, 820	
*Methanofuran*			1		
MfnA: l-Tyrosine decarboxylase		15000		1116	
and estimated 7 unknown proteins		?	(7)	?	
*Methanopterin*			3		
MptA: GTP cyclohydrolase		15740		1196	
MptB: Cyclic phosphodiesterase		02460		1668	
RFAP: Ribofuranosylaminobenzene 5′-P-synthase		12280		830	
and estimated 7 unknown proteins		?	(7)	?	
*Coenzyme M*			5		
ComA: Phosphosulfolactate synthase		02530		1674	
ComB: 2-Phosphosulfolactate phosphatase		15590		1182	
ComC: Sulfolactate dehydrogenase		15830		1205	
ComDE: Sulfopyruvate decarboxylase		15840,15850		1206, 1207	
ComF: Sulfoacetaldehyde dehydrogenase		?	(1)	?	
*Coenzyme B*			4		
LeuA homolog: Isopropylmalate synthase		00630 or 02150		1481 or 1630	
LeuB homolog: Isopropylmalate dehydrogenase		17740 or 06370		1388 or 184	
LeuCD homolog: Isopropylmalate isomerase		02160 or 17720, 12270 or 17730		1386 or 1631, 829 or 1387	
and estimated 2 unknown proteins		?	(2)	?	

*Ion transport systems*					

Ni^2+^ ABC transporter NikMNOQ (=CbiM1N1O1Q1)		02830–02860	4	1704–1707	
Co^2+^ ABC transporter CbiM2N2O2Q2		05800–05830	4	130–133	
Fe^2+^ ABC transporter FeoAB		17520, 17510	2	1362, 1361	
Zn^2+^ ABC transporter ZnuABC/ZupT		09840–09860, 08660	4	604–606, 473	
Mg^2+^ transporter MgtE		10010	1	620	
Ca^2+^ transporter (ATPase)		13830	1	1001	
K^+^ transporter TrkAH		16520, 16510	2	1265, 1264	
MoO_4_^2−^ ABC transporter ModA1B1C1		13080, 13090, 13110	3	920, 921, 924	
WO_4_^2−^ ABC transporter ModA2B2C2		08720–08740	3	477–479	
HPO_4_^2−^ ABC transport system PstABCS1S2/PhoU1U2 Putative transcriptional regulator of the *pst/phu *operon		03020–03070, 03090 03000	8	1727–1732, 1734 1724	

## References

[1] Zeikus JG, Wolfe RS (1972). *Methanobacterium thermoautotrophicus Sp N*, an anaerobic, autotrophic, extreme thermophile. *Journal of Bacteriology*.

[2] Fuchs G, Stupperich E, Thauer RK (1978). Acetate assimilation and the synthesis of alanine, aspartate and glutamate in *Methanobacterium thermoautotrophicum*. *Archives of Microbiology*.

[3] DiMarco AA, Bobik TA, Wolfe RS (1990). Unusual coenzymes of methanogenesis. *Annual Review of Biochemistry*.

[4] Thauer RK (1998). Biochemistry of methanogenesis: a tribute to Marjory Stephenson. *Microbiology*.

[5] Thauer RK, Kaster AK, Goenrich M, Schick M, Hiromoto T, Shima S (2010). Hydrogenases from methanogenic archaea, nickel, a novel cofactor, and H_2_ storage. *Annual Review of Biochemistry*.

[6] Thauer RK, Kaster AK, Seedorf H, Buckel W, Hedderich R (2008). Methanogenic archaea: ecologically relevant differences in energy conservation. *Nature Reviews Microbiology*.

[7] Smith DR, Doucette-Stamm LA, Deloughery C (1997). Complete genome sequence of *Methanobacterium thermoautotrophicum* ΔH: functional analysis and comparative genomics. *Journal of Bacteriology*.

[8] Liesegang H, Kaster A-K, Wiezer A (2010). Complete genome sequence of *Methanothermobacter marburgensis*, a methanoarchaeon model organism. *Journal of Bacteriology*.

[9] Bapteste E, Brochier C, Boucher Y (2005). Higher-level classification of the Archaea: evolution of methanogenesis and methanogens. *Archaea*.

[10] Sakai S, Imachi H, Hanada S, Ohashi A, Harada H, Kamagata Y (2008). *Methanocella paludicola* gen. nov., sp. nov., a methane-producing archaeon, the first isolate of the lineage ’Rice Cluster I’, and proposal of the new archaeal order Methanocellales ord. nov. *International Journal of Systematic and Evolutionary Microbiology*.

[11] Jussofie A, Gottschalk G (1986). Further studies on the distribution of cytochromes in methanogenic bacteria. *FEMS Microbiology Letters*.

[12] Abken HJ, Tietze M, Brodersen J, Bäumer S, Beifuss U, Deppenmeier U (1998). Isolation and characterization of methanophenazine and function of phenazines in membrane-bound electron transport of M*ethanosarcina mazei* Gö1. *Journal of Bacteriology*.

[13] Stock T, Selzer M, Rother M (2010). In vivo requirement of selenophosphate for selenoprotein synthesis in archaea. *Molecular Microbiology*.

[14] Guss AM, Kulkarni G, Metcalf WW (2009). Differences in hydrogenase Gene expression between *Methanosarcina acetivorans* and *Methanosarcina barkeri*. *Journal of Bacteriology*.

[15] Conrad R (2009). The global methane cycle: recent advances in understanding the microbial processes involved. *Environmental Microbiology Reports*.

[16] Conrad R, Erkel C, Liesack W (2006). Rice Cluster I methanogens, an important group of Archaea producing greenhouse gas in soil. *Current Opinion in Biotechnology*.

[17] Wasserfallen A, Nölling J, Pfister P, Reeve J, De Macario EC (2000). Phylogenetic analysis of 18 thermophilic *Methanobacterium* isolates supports the proposals to create a new genus, Methanothermobacter gen. nov., and to reclassify several isolates in three species, *Methanothermobacter thermautotrophicus* comb. nov., *Methanothermobacter wolfeii* comb. nov., and *Methanothermobacter marburgensis* sp. nov. *International Journal of Systematic and Evolutionary Microbiology*.

[18] Battistuzzi FU, Feijao A, Hedges SB (2004). A genomic timescale of prokaryote evolution: insights into the origin of methanogenesis, phototrophy, and the colonization of land. *BMC Evolutionary Biology*.

[19] Brandis A, Thauer RK, Stetter KO (1981). Relatedness of strains ΔH and Marburg of *Methanobacterium thermoautotrophicum*. *Zentralblatt für Bakteriologie, Mikrobiologie und Hygiene, Abt. 1, Originale C*.

[20] Bokranz M, Klein A, Meile L (1990). Complete nucleotide sequenc of plasmid pME2001 of *Methanobacterium thermoautotrophicum* (Marburg). *Nucleic Acids Research*.

[21] Luo Y, Leisinger T, Wasserfallen A (2001). Comparative sequence analysis of plasmids pME2001 and pME2200 of *Methanothermobacter marburgensis* strains Marburg and ZH3. *Plasmid*.

[22] Jordan M, Meile L, Leisinger T (1989). Organization of *Methanobacterium thermoautotrophicum* bacteriophage *ψ*M1 DNA. *Molecular and General Genetics*.

[23] Meile L, Jenal U, Studer D, Jordan M, Leisinger T (1989). Characterization of *ψ*M1, a virulent phage of *Methanobacterium thermoautotrophicum* Marburg. *Archives of Microbiology*.

[24] Nolling J, Groffen A, De Vos WM (1993). ΦF1 and ΦF3, two novel virulent, archaeal phages infecting different thermophilic strains of the genus Methanobacterium. *Journal of General Microbiology*.

[25] Luo Y, Pfister P, Leisinger T, Wasserfallen A (2001). The genome of archaeal prophage *ψ*M100 encodes the lytic enzyme responsible for autolysis of *Methanothermobacter wolfeii*. *Journal of Bacteriology*.

[26] Bertram PA, Schmitz RA, Linder D, Thauer RK (1994). Tungstate can substitute for molybdate in sustaining growth of *Methanobacterium thermoautotrophicum*. Identification and characterization of a tungsten isoenzyme of formylmethanofuran dehydrogenase. *Archives of Microbiology*.

[27] Schoenheit P, Moll J, Thauer RK (1980). Growth parameters (K_S_, *μ*
_max_, Y_S_) of *Methanobacterium thermoautotrophicum*. *Archives of Microbiology*.

[28] Tanner RS, McInerney MJ, Nagle DP (1989). Formate auxotroph of *Methanobacterium thermoautotrophicum* Marburg. *Journal of Bacteriology*.

[29] Oberlies G, Fuchs G, Thauer RK (1980). Acetate thiokinase and the assimilation of acetate in *Methanobacterium thermoautotrophicum*. *Archives of Microbiology*.

[30] Eikmanns B, Jaenchen R, Thauer RK (1983). Propionate assimilation by methanogenic bacteria. *Archives of Microbiology*.

[31] Hüster R, Thauer RK (1983). Pyruvate assimilation by *Methanobacterium thermoautotrophicum*. *FEMS Microbiology Letters*.

[32] Tersteegen A, Linder D, Thauer RK, Hedderich R (1997). Structures and functions of four anabolic 2-oxoacid oxidoreductases in *Methanobacterium thermoautotrophicum*. *European Journal of Biochemistry*.

[33] Diekert G, Gilles HH, Jaenchen R, Thauer RK (1980). Incorporation of 8 succinate per mol nickel into factors F_430_ by *Methanobacterium thermoautotrophicum*. *Archives of Microbiology*.

[34] Diekert G, Jaenchen R, Thauer RK (1980). Biosynthetic evidence for a nickel tetrapyrrole structure of factor F_430_ from *Methanobacterium thermoautotrophicum*. *FEBS Letters*.

[35] Jaenchen R, Diekert G, Thauer RK (1981). Incorporation of methionine-derived methyl groups into factor F_430_ by *Methanobacterium thermoautotrophicum*. *FEBS Letters*.

[36] Jaenchen R, Schonheit P, Thauer RK (1984). Studies on the biosynthesis of coenzyme F_420_ in methanogenic bacteria. *Archives of Microbiology*.

[37] Mukhopadhyay B, Stoddard SF, Wolfe RS (1998). Purification, regulation, and molecular and biochemical characterization of pyruvate carboxylase from *Methanobacterium thermoautotrophicum* strain ΔH. *Journal of Biological Chemistry*.

[38] Nolling J, Hahn D, Ludwig W, De Vos WM (1993). Phylogenetic analysis of thermophilic *Methanobacterium Sp*—evidence for a formate-utilizing ancestor. *Systematic and Applied Microbiology*.

[39] Haft DH, Self WT (2008). Orphan SelD proteins and selenium-dependent molybdenum hydroxylases. *Biology Direct*.

[40] Itoh Y, Sekine SI, Matsumoto E (2009). Structure of selenophosphate synthetase essential for selenium incorporation into proteins and RNAs. *Journal of Molecular Biology*.

[41] Leahy SC, Kelly WJ, Altermann E (2010). The genome sequence of the rumen methanogen *Methanobrevibacter ruminantium* reveals new possibilities for controlling ruminant methane emissions. *PloS ONE*.

[42] Ding X, Yang WJ, Min H, Peng XT, Zhou HY, Lu ZM (2010). Isolation and characterization of a new strain of *Methanothermobacter marburgensis* DX01 from hot springs in China. *Anaerobe*.

[43] Needleman SB, Wunsch CD (1970). A general method applicable to the search for similarities in the amino acid sequence of two proteins. *Journal of Molecular Biology*.

[44] Cheng S, Xing D, Call DF, Logan BE (2009). Direct biological conversion of electrical current into methane by electromethanogenesis. *Environmental Science and Technology*.

[45] Villano M, Aulenta F, Ciucci C, Ferri T, Giuliano A, Majone M (2010). Bioelectrochemical reduction of CO_2_ to CH_4_ via direct and indirect extracellular electron transfer by a hydrogenophilic methanogenic culture. *Bioresource Technology*.

[46] Nealson KH (2010). Geomicrobiology: sediment reactions defy dogma. *Nature*.

[47] Nielsen LP, Risgaard-Petersen N, Fossing H, Christensen PB, Sayama M (2010). Electric currents couple spatially separated biogeochemical processes in marine sediment. *Nature*.

[48] Reguera G, McCarthy KD, Mehta T, Nicoll JS, Tuominen MT, Lovley DR (2005). Extracellular electron transfer via microbial nanowires. *Nature*.

[49] Thoma C, Frank M, Rachel R (2008). The Mth60 fimbriae of *Methanothermobacter thermoautotrophicus* are functional adhesins. *Environmental Microbiology*.

[50] Friedrich MW (2005). Methyl-coenzyme M reductase genes: unique functional markers for methanogenic and anaerobic methane-oxidizing Archaea. *Methods in Enzymology*.

[51] Kurtz S, Phillippy A, Delcher AL (2004). Versatile and open software for comparing large genomes. *Genome Biology*.

[52] Vermeij P, Van Der Steen RJT, Keltjens JT, Vogels GD, Leisinger T (1996). Coenzyme F_390_ synthetase from *Methanobacterium thermoautotrophicum* Marburg belongs to the superfamily of adenylate-forming enzymes. *Journal of Bacteriology*.

[53] Filée J, Siguier P, Chandler M (2007). Insertion sequence diversity in Archaea. *Microbiology and Molecular Biology Reviews*.

[54] Deveau H, Garneau JE, Moineau S (2010). CRISPR/Cas system and its role in phage-bacteria interactions. *Annual Review of Microbiology*.

[55] Karginov FV, Hannon GJ (2010). The CRISPR system: small RNA-guided defense in bacteria and archaea. *Molecular Cell*.

[56] Marraffini LA, Sontheimer EJ (2010). Self versus non-self discrimination during CRISPR RNA-directed immunity. *Nature*.

[57] Lillestøl RK, Redder P, Garrett RA, Brügger K (2006). A putative viral defence mechanism in archaeal cells. *Archaea*.

[58] Berg IA, Kockelkorn D, Ramos-Vera WH (2010). Autotrophic carbon fixation in archaea. *Nature Reviews Microbiology*.

[59] de Poorter LMI, Geerts WJ, Keltjens JT (2005). Hydrogen concentrations in methane-forming cells probed by the ratios of reduced and oxidized coenzyme F_420_. *Microbiology*.

[60] Kaster AK, Moll J, Parey K, Thauer R (2011). Coupling of ferredoxin- and heterodisulfide reduction with H_2_ via electron bifurcation in hydrogenotrophic methanogenic archaea. *Proceedings of the National Academy of Sciences of the United States of America*.

[61] Bertram PA, Thauer RK (1994). Thermodynamics of the formylmethanofuran dehydrogenase reaction in *Methanobacterium thermoautotrophicum*. *European Journal of Biochemistry*.

[62] Costa KC, Wong PM, Wang T (2010). Protein complexing in a methanogen suggests electron bifurcation and electron delivery from formate to heterodisulfide reductase. *Proceedings of the National Academy of Sciences of the United States of America*.

[63] Nolling J, Pihl TD, Vriesema A, Reeve JN (1995). Organization and growth phase-dependent transcription of methane genes in two regions of the *Methanobacterium thermoautotrophicum* genome. *Journal of Bacteriology*.

[64] Pihl TD, Sharma S, Reeve JN (1994). Growth phase-dependent transcription of the genes that encode the two methyl coenzyme M reductase isoenzymes and *N*
^5^-methyltetrahydromethanopterin:Coenzyme M methyltransferase in *Methanobacterium thermoautotrophicum* ΔH. *Journal of Bacteriology*.

[65] Hamann N, Mander GJ, Shokes JE, Scott RA, Bennati M, Hedderich R (2007). A cysteine-rich CCG domain contains a novel [4Fe-4S] cluster binding motif as deduced from studies with subunit B of heterodisulfide reductase from *Methanothermobacter marburgensis*. *Biochemistry*.

[66] Hedderich R, Koch J, Linder D, Thauer RK (1994). The heterodisulfide reductase from *Methanobacterium thermoautotrophicum* contains sequence motifs characteristic of pyridine-nucleotide-dependent thioredoxin reductases. *European Journal of Biochemistry*.

[67] Anderson I, Ulrich LE, Lupa B (2009). Genomic characterization of methanomicrobiales reveals three classes of methanogens. *PLoS ONE*.

[68] Böck A, King PW, Blokesch M, Posewitz MC (2006). Maturation of Hydrogenases. *Advances in Microbial Physiology*.

[69] Afting C, Hochheimer A, Thauer RK (1998). Function of H-forming methylenetetrahydromethanopterin dehydrogenase from *Methanobacterium thermoautotrophicum* in coenzyme F_420_ reduction with H_2_. *Archives of Microbiology*.

[70] Hendrickson EL, Leigh JA (2008). Roles of coenzyme F_420_-reducing hydrogenases and hydrogen- and F_420_-dependent methylenetetrahydromethanopterin dehydrogenases in reduction of F_420_ and production of hydrogen during methanogenesis. *Journal of Bacteriology*.

[71] Lupa B, Hendrickson EL, Leigh JA, Whitman WB (2008). Formate-dependent H_2_production by the mesophilic methanogen *Methanococcus maripaludis*. *Applied and Environmental Microbiology*.

[72] Welte C, Kallnik V, Grapp M, Bender G, Ragsdale S, Deppenmeier U (2010). Function of Ech hydrogenase in ferredoxin-dependent, membrane-bound electron transport in *Methanosarcina mazei*. *Journal of Bacteriology*.

[73] Major TA, Liu Y, Whitman WB (2010). Characterization of energy-conserving hydrogenase B in *Methanococcus maripaludis*. *Journal of Bacteriology*.

[74] Porat I, Kim W, Hendrickson EL (2006). Disruption of the operon encoding Ehb hydrogenase limits anabolic CO_2_ assimilation in the archaeon *Methanococcus maripaludis*. *Journal of Bacteriology*.

[75] Tersteegen A, Hedderich R (1999). *Methanobacterium thermoautotrophicum* encodes two multisubunit membrane- bound [NiFe] hydrogenases. Transcription of the operons and sequence analysis of the deduced proteins. *European Journal of Biochemistry*.

[76] Hedderich R, Forzi L (2006). Energy-converting [NiFe] hydrogenases: more than just H_2_activation. *Journal of Molecular Microbiology and Biotechnology*.

[77] Vorholt JA, Thauer RK (2002). Molybdenum and tungsten enzymes in C1 metabolism. *Metal Ions in Biological Systems*.

[78] Hochheimer A, Hedderich R, Thauer RK (1998). The formylmethanofuran dehydrogenase isoenzymes in *Methanobacterium wolfei* and *Methanobacterium thermoautotrophicum*: induction of the molybdenum isoenzyme by molybdate and constitutive synthesis of the tungsten isoenzyme. *Archives of Microbiology*.

[79] Hochheimer A, Linder D, Thauer RK, Hedderich R (1996). The molybdenum formylmethanofuran dehydrogenase operon and the tungsten formylmethanofuran dehydrogenase operon from *Methanobacterium thermoautotrophicum*. Structures and transcriptional regulation. *European Journal of Biochemistry*.

[80] Grabarse W, Vaupel M, Vorholt JA (1999). The crystal structure of methenyltetrahydromethanopterin cyclohydrolase from the hyperthermophilic archaeon *Methanopyrus kandleri*. *Structure*.

[81] Shima S, Warkentin E, Thauer RK, Ermler U (2002). Structure and function of enzymes involved in the methanogenic pathway utilizing carbon dioxide and molecular hydrogen. *Journal of Bioscience and Bioengineering*.

[82] Buchenau B, Thauer RK (2004). Tetrahydrofolate-specific enzymes in *Methanosarcina barkeri* and growth dependence of this methanogenic archaeon on folic acid or p-aminobenzoic acid. *Archives of Microbiology*.

[83] Gottschalk G, Thauer RK (2001). The Na^+^-translocating methyltransferase complex from methanogenic archaea. *Biochimica et Biophysica Acta*.

[84] Shin DH (2008). Preliminary structural studies on the MtxX protein from *Methanococcus jannaschii*. *Acta Crystallographica Section D: Biological Crystallography*.

[85] Stroup D, Reeve JN (1993). Identification of the *mcrC* gene product in *Methanococcus vannielii*. *FEMS Microbiology Letters*.

[86] Kuhner CH, Lindenbach BD, Wolfe RS (1993). Component A2 of methylcoenzyme M reductase system from *Methanobacterium thermoautotrophicum* ΔH: nucleotide sequence and functional expression by *Escherichia coli*. *Journal of Bacteriology*.

[87] Hedderich R, Albracht SPJ, Linder D, Koch J, Thauer RK (1992). Isolation and characterization of polyferredoxin from *Methanobacterium thermoautotrophicum*. The *mvhB* gene product of the methylviologen-reducing hydrogenase operon. *FEBS Letters*.

[88] Lienard T, Becher B, Marschall M, Bowien S, Gottschalk G (1996). Sodium ion translocation by N-methyltetrahydromethanopterin:coenzyme M methyltransferase from *Methanosarcina mazei* Gö1 reconstituted in ether lipid liposomes. *European Journal of Biochemistry*.

[89] Pisa KY, Huber H, Thomm M, Müller V (2007). A sodium ion-dependent AA ATP synthase from the hyperthermophilic archaeon *Pyrococcus furiosus*. *FEBS Journal*.

[90] Vidová M, Šmigáň P (2010). Unique structural and functional properties of AA ATPase/synthase from archaeaUnikátne štruktúrne a funkčné vlastnosti AA ATPáz/syntáz Z archaea. *Chemicke Listy*.

[91] Šurín S, Čuboňová L, Majerník AI, McDermott P, Chong JPJ, Šmigáň P (2007). Isolation and characterization of an amiloride-resistant mutant of *Methanothermobacter thermautotrophicus* possessing a defective Na^+^/H^+^ antiport. *FEMS Microbiology Letters*.

[92] McGlynn SE, Boyd ES, Shepard EM (2010). Identification and characterization of a novel member of the radical AdoMet enzyme superfamily and implications for the biosynthesis of the Hmd hydrogenase active site cofactor. *Journal of Bacteriology*.

[93] Shima S, Thauer RK (2007). A third type of hydrogenase catalyzing H_2_activation. *Chemical Record*.

[94] Schoenheit P, Moll J, Thauer RK (1979). Nickel, cobalt, and molybdenum requirement for growth of *Methanobacterium thermoautotrophicum*. *Archives of Microbiology*.

[95] Vinella D, Brochier-Armanet C, Loiseau L, Talla E, Barras F (2009). Iron-sulfur (Fe/S) protein biogenesis: phylogenomic and genetic studies of A-type carriers. *PLoS Genetics*.

[96] Boyd JM, Drevland RM, Downs DM, Graham DE (2009). Archaeal ApbC/Nbp35 homologs function as iron-sulfur cluster carrier proteins. *Journal of Bacteriology*.

[97] Boyd JM, Sondelski JL, Downs DM (2009). Bacterial apbC protein has two biochemical activities that are required for *in vivo* function. *Journal of Biological Chemistry*.

[98] Liu Y, Sieprawska-Lupa M, Whitman WB, White RH (2010). Cysteine is not the sulfur source for iron-sulfur cluster and methionine biosynthesis in the methanogenic archaeon *Methanococcus maripaludis*. *Journal of Biological Chemistry*.

[99] Schwarz G, Mendel RR, Ribbe MW (2009). Molybdenum cofactors, enzymes and pathways. *Nature*.

[100] Krautler B, Moll J, Thauer RK (1987). The corrinoid from *Methanobacterium thermoautotrophicum* (Marburg strain). Spectroscopic structure analysis and identifcation as Co(*β*)-cyano-5’-hydroxybenzimidazolyl-cobamide (factor III). *European Journal of Biochemistry*.

[101] Frank S, Deery E, Brindley AA (2007). Elucidation of substrate specificity in the cobalamin (vitamin B_12_ ) biosynthetic methyltransferases: structure and function of the C20 methyltransferase (CbiL) from *Methanothermobacter thermautotrophicus*. *Journal of Biological Chemistry*.

[102] Rodionov DA, Vitreschak AG, Mironov AA, Gelfand MS (2003). Comparative genomics of the vitamin B12 metabolism and regulation in prokaryotes. *Journal of Biological Chemistry*.

[103] Sauer K, Thauer RK, Banerjee R (1999). The role of corrinoids in methanogenesis. *Chemistry and Biochemistry of B12*.

[104] Woodson JD, Escalante-Semerena JC (2004). CbiZ, an amidohydrolase enzyme required for salvaging the coenzyme B precursor cobinamide in archaea. *Proceedings of the National Academy of Sciences of the United States of America*.

[105] Woodson JD, Escalante-Semerena JC (2006). The *cbiS* gene of the archaeon *Methanopyrus kandleri* AV19 encodes a bifunctional enzyme with adenosylcobinamide amidohydrolase and *α*-ribazole-phosphate phosphatase activities. *Journal of Bacteriology*.

[106] Woodson JD, Zayas CL, Escalante-Semerena JC (2003). A new pathway for salvaging the coenzyme B12 precursor cobinamide in archaea requires cobinamide-phosphate synthase (CbiB) enzyme activity. *Journal of Bacteriology*.

[107] Johnson EF, Mukhopadhyay B (2005). A new type of sulfite reductase, a novel coenzyme F_420_-dependent enzyme, from the methanarchaeon *Methanocaldococcus jannaschii*. *Journal of Biological Chemistry*.

[108] Pfaltz A, Kobelt A, Hunster R, Thauer RK (1988). Biosynthesis of coenzyme F_430_ in methanogenic bacteria. Identification of 15,17^3^-seco-F_430_-17^3^-acid as an intermediate. *European Journal of Biochemistry*.

[109] Lundqvist J, Elmlund D, Heldt D (2009). The AAA(+) motor complex of subunits CobS and CobT of cobaltochelatase visualized by single particle electron microscopy. *Journal of Structural Biology*.

[110] Staples CR, Lahiri S, Raymond J, Von Herbulis L, Mukhophadhyay B, Blankenship RE (2007). Expression and association of group IV nitrogenase NifD and NifH homologs in the non-nitrogen-fixing archaeon *Methanocaldococcus jannaschii*. *Journal of Bacteriology*.

[111] Muraki N, Nomata J, Ebata K (2010). X-ray crystal structure of the light-independent protochlorophyllide reductase. *Nature*.

[112] Meyerdierks A, Kube M, Kostadinov I (2010). Metagenome and mRNA expression analyses of anaerobic methanotrophic archaea of the ANME-1 group. *Environmental Microbiology*.

[113] Mayr S, Latkoczy C, Krüger M (2008). Structure of an F_430_ variant from archaea associated with anaerobic oxidation of methane. *Journal of the American Chemical Society*.

[114] Thauer RK, Shima S (2008). Methane as fuel for anaerobic microorganisms. *Annals of the New York Academy of Sciences*.

[115] Vandevenne M, Filee P, Scarafone N (2007). The *Bacillus licheniformis* BlaP *β*-lactamase as a model protein scaffold to study the insertion of protein fragments. *Protein Science*.

[116] Graham DE, White RH (2002). Elucidation of methanogenic coenzyme biosyntheses: from spectroscopy to genomics. *Natural Product Reports*.

[117] Forouhar F, Abashidze M, Xu H (2008). Molecular insights into the biosynthesis of the F_420_ coenzyme. *Journal of Biological Chemistry*.

[118] Grochowski LL, Xu H, White RH (2008). Identification and characterization of the 2-phospho-L-lactate guanylyltransferase involved in coenzyme F_420_ biosynthesis. *Biochemistry*.

[119] Grochowski LL, Xu H, White RH (2006). Identification of lactaldehyde dehydrogenase in *Methanocaldococcus jannaschii* and its involvement in production of lactate for F_420_ biosynthesis. *Journal of Bacteriology*.

[120] Nocek B, Evdokimova E, Proudfoot M (2007). Structure of an amide bond forming F_420_:gamma-glutamyl ligase from *Archaeoglobus fulgidus*—a member of a new family of non-ribosomal peptide synthases. *Journal of Molecular Biology*.

[121] Li H, Xu H, Graham DE, White RH (2003). Glutathione synthetase homologs encode *α*-L-glutamate ligases for methanogenic coenzyme F_420_ and tetrahydrosarcinapterin biosyntheses. *Proceedings of the National Academy of Sciences of the United States of America*.

[122] Seedorf H, Dreisbach A, Hedderich R, Shima S, Thauer RK (2004). F_420_H_2_ oxidase (FprA) from *Methanobrevibacter arboriphilus*, a coenzyme F_420_-dependent enzyme involved in O_2_ detoxification. *Archives of Microbiology*.

[123] Ceh K, Demmer U, Warkentin E (2009). Structural basis of the hydride transfer mechanism in F_420_-dependent methylenetetrahydromethanopterin dehydrogenase. *Biochemistry*.

[124] Seedorf H, Hagemeier CH, Shima S, Thauer RK, Warkentin E, Ermler U (2007). Structure of coenzyme F_420_H_2_ oxidase (FprA), a di-iron flavoprotein from methanogenic Archaea catalyzing the reduction of O_2_ to H_2_O. *FEBS Journal*.

[125] Johnson EF, Mukhopadhyay B, Friedrich CDACG (2008). A novel coenzyme F_420_ dependent sulfite reductase and a small sulfite reductase in methanogenic archaea. *Microbial Sulfur Metabolism*.

[126] Raemakers-Franken PC, Brand RJM, Kortstee AJ, Van der Drift C, Vogels GD (1991). Ammonia assimilation and glutamate incorporation in coenzyme F_420_ derivatives of *Methanosarcina barkeri*. *Antonie van Leeuwenhoek, International Journal of General*.

[127] White WB, Ferry JG (1992). Identification of formate dehydrogenase-specific mRNA species and nucleotide sequence of the fdhC gene of *Methanobacterium formicicum*. *Journal of Bacteriology*.

[128] Ownby K, Xu H, White RH (2005). A *Methanocaldococcus jannaschii* archaeal signature gene encodes for a 5-formaminoimidazole-4-carboxamide-1-*β*-D-ribofuranosyl 5′- monophosphate synthetase: a new enzyme in purine biosynthesis. *Journal of Biological Chemistry*.

[129] Waight AB, Love J, Wang DAN (2010). Structure and mechanism of a pentameric formate channel. *Nature Structural &amp; Molecular Biology*.

[130] Wood GE, Haydock AK, Leigh JA (2003). Function and regulation of the formate dehydrogenase genes of the methanogenic archaeon *Methanococcus maripaludis*. *Journal of Bacteriology*.

[131] Kezmarsky ND, Xu H, Graham DE, White RH (2005). Identification and characterization of a L-tyrosine decarboxylase in *Methanocaldococcus jannaschii*. *Biochimica et Biophysica Acta*.

[132] Mashhadi Z, Xu H, White RH (2009). An Fe^2+^-dependent cyclic phosphodiesterase catalyzes the hydrolysis of 7,8-dihydro-D-neopterin 2′,3′-cyclic phosphate in methanopterin biosynthesis. *Biochemistry*.

[133] Bechard ME, Garcia D, Greene D, Rasche ME Overproduction, characterization, and site-directed mutagenisis of RFAP synthase from the methanogen *Methanothermobacter thermoautotrophicus delta H*.

[134] Li F, Hagemeier CH, Seedorf H, Gottschalk G, Thauer RK (2007). Re-citrate synthase from *Clostridium kluyveri* is phylogenetically related to homocitrate synthase and isopropylmalate synthase rather than to Si-citrate synthase. *Journal of Bacteriology*.

[135] Drevland RM, Jia Y, Palmer DRJ, Graham DE (2008). Methanogen homoaconitase catalyzes both hydrolyase reactions in coenzyme B biosynthesis. *Journal of Biological Chemistry*.

[136] Williams RJP, Frausto da Silva JJR (1996). *The Natural Selection of the Chemical Elements*.

[137] Zhang Y, Rodionov DA, Gelfand MS, Gladyshev VN (2009). Comparative genomic analyses of nickel, cobalt and vitamin B_12_ utilization. *BMC Genomics*.

[138] Kammler M, Schon C, Hantke K (1993). Characterization of the ferrous iron uptake system of *Escherichia coli*. *Journal of Bacteriology*.

[139] Wang SC, Dias AV, Zamble DB (2009). The “metallo-specific” response of proteins: a perspective based on the *Escherichia coli* transcriptional regulator NikR. *Dalton Transactions*.

[140] Leitch S, Bradley MJ, Rowe JL, Chivers PT, Maroney MJ (2007). Nickel-specific response in the transcriptional regulator, *Escherichia coli* NikR. *Journal of the American Chemical Society*.

[141] Hattori M, Iwase N, Furuya N (2009). Mg^2+^-dependent gating of bacterial MgtE channel underlies Mg^2+^ homeostasis. *EMBO Journal*.

[142] Vanček M, Vidová M, Majerník AI, Šmigáň P (2006). Methanogenesis is Ca^2+^dependent in *Methanothermobacter thermautotrophicus* strain ΔH. *FEMS Microbiology Letters*.

[143] De Hertogh B, Lantin AC, Baret PV, Goffeau A (2004). The archaeal P-type ATPases. *Journal of Bioenergetics and Biomembranes*.

[144] Schonheit P, Beimborn DB, Perski HJ (1984). Potassium accumulation in growing *Methanobacterium thermoautotrophicum* and its relation to the electrochemical proton gradient. *Archives of Microbiology*.

[145] Glasemacher J, Siebers A, Altendorf K, Schönheit P (1996). Low-affinity potassium uptake system in the archaeon *Methanobacterium thermoautotrophicum*: overproduction of a 31-kilodalton membrane protein during growth on low-potassium medium. *Journal of Bacteriology*.

[146] Self WT, Grunden AM, Hasona A, Shanmugam KT (2001). Molybdate transport. *Research in Microbiology*.

[147] Zhang Y, Gladyshev VN (2008). Molybdoproteomes and evolution of molybdenum utilization. *Journal of Molecular Biology*.

[148] Bevers LE, Hagedoorn PL, Krijger GC, Hagen WR (2006). Tungsten transport protein A (WtpA) in *Pyrococcus furiosus*: the first member of a new class of tungstate and molybdate transporters. *Journal of Bacteriology*.

[149] Gerber S, Comellas-Bigler M, Goetz BA, Locher KP (2008). Structural basis of trans-inhibition in a molybdate/tungstate ABC transporter. *Science*.

[150] Aguena M, Spira B (2009). Transcriptional processing of the pst operon of *Escherichia coli*. *Current Microbiology*.

[151] Afting C, Kremmer E, Brucker C, Hochheimer A, Thauer RK (2000). Regulation of the synthesis of H_2_-forming methylenetetrahydromethanopterin dehydrogenase (Hmd) and of HmdII and HmdIII in *Methanothermobacter marburgensis*. *Archives of Microbiology*.

[152] Osman D, Cavet JS (2010). Bacterial metal-sensing proteins exemplified by ArsR-SmtB family repressors. *Natural Product Reports*.

[153] Hochheimer A, Hedderich R, Thauer RK (1999). The DNA binding protein Tfx from *Methanobacterium thermoautotrophicum*: structure, DNA binding properties and transcriptional regulation. *Molecular Microbiology*.

[154] Bonacker LG, Baudner S, Thauer RK (1992). Differential expression of the two methyl-coenzyme M reductases in *Methanobacterium thermoautotrophicum* as determined immunochemically via isoenzyme-speficic antisera. *European Journal of Biochemistry*.

[155] Morgan RM, Pihl TD, Nölling J, Reeve JN (1997). Hydrogen regulation of growth, growth yields, and methane gene transcription in *Methanobacterium thermoautotrophicumδ*H. *Journal of Bacteriology*.

[156] Shinzato N, Enoki M, Sato H, Nakamura K, Matsui T, Kamagata Y (2008). Specific DNA binding of a potential transcriptional regulator, inosine 5′-monophosphate dehydrogenase-related protein VII, to the promoter region of a methyl coenzyme M reductase I-encoding operon retrieved from *Methanothermobacter thermautotrophicus* strain ΔH. *Applied and Environmental Microbiology*.

[157] Lenz O, Bernhard M, Buhrke T, Schwartz E, Friedrich B (2002). The hydrogen-sensing apparatus in *Ralstonia eutropha*. *Journal of Molecular Microbiology and Biotechnology*.

[158] Leigh JA, Dodsworth JA (2007). Nitrogen regulation in bacteria and archaea. *Annual Review of Microbiology*.

[159] Lie TJ, Hendrickson EL, Niess UM, Moore BC, Haydock AK, Leigh JA (2010). Overlapping repressor binding sites regulate expression of the *Methanococcus maripaludis* glnK operon. *Molecular Microbiology*.

[160] Ermler U, Grabarse W, Shima S, Goubeaud M, Thauer RK (1997). Crystal structure of methyl-coenzyme M reductase: the key enzyme of biological methane formation. *Science*.

[161] Kahnt J, Buchenau B, Mahlert F, Krüger M, Shima S, Thauer RK (2007). Post-translational modifications in the active site region of methyl-coenzyme M reductase from methanogenic and methanotrophic archaea. *FEBS Journal*.

[162] Selmer T, Kahnt J, Goubeaud M (2000). The biosynthesis of methylated amino acids in the active site region of methyl-coenzyme M reductase. *Journal of Biological Chemistry*.

[163] Yan F, Lamarre JM, Röhrich R (2010). RImN and Cfr are Radical SAM Enzymes Involved in Methylation of Ribosomal RNA. *Journal of the American Chemical Society*.

[164] Graham DE, Bock CL, Schalk-Hihi C, Lu ZJ, Markham GD (2000). Identification of a highly diverged class of S-adenosylmethionine synthetases in the archaea. *Journal of Biological Chemistry*.

[165] Klenk HP, Clayton RA, Tomb JF (1997). The complete genome sequence of the hyperthermophilic, sulphate-reducing archaeon *Archaeoglobus fulgidus*. *Nature*.

[166] Strittmatter AW, Liesegang H, Rabus R (2009). Genome sequence of *Desulfobacterium autotrophicum* HRM2, a marine sulfate reducer oxidizing organic carbon completely to carbon dioxide. *Environmental Microbiology*.

[167] Chistoserdova L, Vorholt JA, Thauer RK, Lidstrom ME (1998). C1 transfer enzymes and coenzymes linking methylotrophic bacteria and methanogenic archaea. *Science*.

[168] Graf EG, Thauer RK (1981). Hydrogenase from *Methanobacterium thermoautotrophicum*, a nickel-containing enzyme. *FEBS Letters*.

[169] Forzi L, Sawers RG (2007). Maturation of [NiFe]-hydrogenases in *Escherichia coli*. *BioMetals*.

