# Supplementary material for: More Than 200 Genes Required for Methane Formation from H2 and CO2 and Energy Conservation Are Present in Methanothermobacter marburgensis and Methanothermobacter thermautotrophicus
Source: Archaea. 2011 Apr 27;2011:973848. doi: 10.1155/2011/973848 (PMC3087415; doi:10.1155/2011/973848)
Supplement: Supplementary file 1 — The methods of sequencing employed and the data basis used in the sequence comparisons are described. A list of methanogen specific genes is given (supplementary Table 1). It is described how the CDS not in common were traced back to putative gene splitting events, gene deletion events, gene duplication events and lateral gene transfer events. For Methanothermobacter marburgensis, CDS not in common are listed in supplementary Table 2 and those for M. thermoautotrophicus in supplementary Table 3. [file 973848.f1.pdf]

More than 200 genes required for methane formation from H_2 and CO_2 and energy conservation in *Methanothermobacter marburgensis* and *M. thermautotrophicus* (21-02-11)

Anne-Kristin Kaster[1], Meike Goenrich[1], Henning Seedorf [2], Heiko Liesegang[3], Antje Wollherr[3], Gerhard Gottschalk[3], and Rudolf K. Thauer[1]*

[1] Max Planck Institute for Terrestrial Microbiology, 35043 Marburg, Germany

[2] Center for Genome Sciences and Systems Biology, Washington University School of Medicine, St. Louis MO 63108, USA

[3] Göttingen Genomics Laboratory, Institute of Microbiology and Genetics, Georg August University, 37077 Göttingen, Germany

* Corresponding author: Prof. Dr. R. Thauer (thauer@mpi-marburg.mpg.de

Sequencing

Sequencing of the *M. marburgensis* genome was done in a combined Sanger/454-Pyrosequencing approach. 454 sequencing resulted in 216,097 reads giving ~52 Mbp sequencing data corresponding to a 32X coverage. In addition 6,000 genomic fragments were cloned into the TOPO TA vector (Quiagen, Hilden) and sequenced on a ABI 3730 capillar sequencer from both ends. The ABI sequences resulted in 11808 reads corresponding to an additional coverage of 7.4X. Remaining gaps were closed by sequences generated on gap spanning PCR products by an ABI 3730 capillary sequencer. The overall quality was set to a minimum confidence of PHRED 45 for the complete genome [1].

Data basis

The data basis for the comparison comprised the genome sequences of the following Archaea: *Methanothermobacter marburgensis* (NC_014408/CP001710), *Methanothermobacter thermautotrophicus* (NC_000916), *Methanosphaera stadtmanae* (NC_007681), *Methanobrevibacter smithii* ATCC35061 (NC_009515),

Methanobrevibacter ruminantium M1 (NC_013790), *Methanocaldococcus jannaschii* (NC_000909), *Methanococcus aeolicus* Nankai-3 (NC_009635), *Methanococcus maripaludis* S2 (NC_005791), *Methanococcus vannielii* SB (NC_009634), *Methanocorpusculum labreanum* Z (NC_008942), *Methanopyrus kandleri* AV19 (NC_003551), *Methanosarcina mazei* Gö1 (NC_003901), *Methanosarcina acetivorans* C2A (NC_993552), *Methanosarcina barkeri* Fusaro (NC_007355), *Archaeoglobus fulgidus* (NC_000917), *Hyperthermus butylics* (NC_008818), *Picrophilus torridus* (AE017261), *Pyrobaculum aerophilum* IM2 (NC_003364), *Sulfolobus solfataricus* P2 (NC_002754), *Thermoplasma acidophilum* (NC_002578), *Halobacterium sp.* NRC-1 (NC_002607) and *Methanocella paludicula gen. Nov., sp. nov.* (formally "uncultured methanogenic archaeon RC-I") (AM114193). Where indicated, genome comparisons were extended to sequences of other Archaea, Bacteria and Eukarya from the NCBI data bases. The comparisons were completed in May 2010.

Methanogen specific CDS

In the methanogens sequenced to date there are 27 CDS and 7 homologous pairs of CDS found in every methanogen but not in non methanogens. These are listed in Supplementary Table 1.

CDS not in common

In order to trace back the origin of the CDS in *M. marburgensis* and *M. thermautotrophicus* not in common we looked for all homologous sequences in the two genomes and whether the homologs are also found in the genomes of other Archaea. Based on this analysis the CDS not in common are interpreted to be the result of gene splitting (frame shifts caused by single base insertion/deletion), gene deletion-, gene duplication-, and lateral gene transfer events that have occurred in only one of the two organisms since they descended from their most recent common ancestor.

Putative gene splitting events: Of the 145 CDS in *M. marburgensis* not in common (Supplementary Table 2) there are 22 CDS with hits to CDS in *M. thermautotrophicus* that themselves show a best hit to another CDS in *M. marburgensis*. These CDS not in

common all turned out to be the result of gene splitting events. Examples are the split genes for one of the ribosome proteins (MTBMA_c05290 + MTBMA_c05280 = MTH 39), for a nucleoside-diphosphate-sugar transferase (MTBMA_c03360 + 03350 = MTH1759), for subunit D of pyruvate synthase (MTBMA_c03160 + MTBMA_c03150 = MTH1740), for a histone acetyltransferase (MTBMA_c12130 + MTBMA_c12140 = MTH817), for subunit A of 2-oxoisovalerate synthase (MTBMA_c10930 + MTBMA_c10920 = MTH705), for a cobalto chelatase (MTBMA_c10570 + MTBMA_c10560 + MTBMA_c10550 = MTH673), and for adenosylcobalamin dependent ribonucleotide-diphosphate reductase (MTBMA_c10320 + MTBMA_c10330 = MTH652). The split of proteins in two is not necessarily associated with a loss of function as evidenced for F_{420}-non-reducing hydrogenase from *Methanococcus voltae* [2] and for a tRNA from *Nanoarchaeum equitans* [3]. In *M. thermautotrophicus* 46 of the 266 CDS not in common belong to split genes (Supplementary Table 3). It has to be considered that of the annotated “split genes” some may be the result of sequencing errors.

Putative gene deletion events: In *M. marburgensis* 44 of the 145 CDS not in common (Supplementary Table 2) are hypothetical proteins that do not show sequence similarities to other CDS in *M. marburgensis*, to any CDS in *M. thermautotrophicus* and to CDS in any other organisms. *Vice versa*, in *M. thermautotrophicus* 67 of the 266 CDS not in common (Supplementary Table 3) are for hypothetical proteins that do not show sequence similarity to other CDS in *M. thermautotrophicus*, to any CDS in *M. marburgensis* or to any CDS in other organism. These 111 (44 + 67) CDS not in common are therefore most probably the results of gene deletion events in the two organisms. The genomes of *M. marburgensis* and *M. thermautotrophicus* have 177 CDS in common that are not found in any other organisms. Therefore, the most recent common ancestor of the two *Methanothermobacter* species must have had at least 288 (177 + 111) ancestor-specific CDS.

Putative gene duplication events: In *M. marburgensis* 44 of the 145 CDS not in common (Supplementary Table 2) only show sequence similarity to other CDS present in *M. marburgensis* indicating that these 44 CDS were generated by gene duplication or

partial gene duplication events. Five of these are associated with IS-like elements, 32 of the 44 CDS are proteins with annotated function, 10 CDS are hypothetical proteins and 2 are conserved hypothetical proteins. In *M. thermautotrophicus* there are 80 CDS not in common that only show sequence similarities to other CDS in *M. thermautotrophicus* (Supplementary Table 3).

In *M. thermautotrophicus* one of the gene duplicates, namely that for an annotated cation-transporting P-ATPase PacL (MTH1001 ≙ MTH481 + MTH482), might have led to an uninhibited membrane-associated ATPase which could explain why the membrane fraction of *M. thermautotrophicus* exhibits much higher ATPase activity than the membrane fraction of *M. marburgensis*. Of possible functional interest is also that in *M. marburgensis* the genes for CdhA (MTBMA_c14220 + 14210 + 14200 ≙ MTBMA_c02870) and CdhB (MTBMA_c14190 ≙ MTBMA_c02880) of the acetyl-CoA synthase/CO dehydrogenase complex are duplicated. It therefore could be possible that in *M. marburgensis* part of the acetyl-CoA synthase/decarbonylase complex, one of the key enzyme complexes involved in CO_2 assimilation, is existing twice, contributing to the ability of *M. marburgensis* to grow more rapidly or to regulate CO_2 fixation differently than *M. thermautotrophicus*. However, expression data supporting this contention are not available.

Putative lateral gene transfer events: In *M. marburgensis* 35 of the 145 CDS not in common and in *M. thermautotrophicus* 72 of the 266 CDS not in common do not show sequence similarity to any CDS in *M. marburgensis* or *M. thermautotrophicus* but to CDS in other achaea and bacteria and are therefore considered to have been acquired via lateral gene transfer (Supplementary Tables 2 and 3): . In *M. marburgensis*, amongst the 35 CDS are 2 CDS (MTBMA_c01250 and MTBMA_c01240) for a putative transposase which is functionally associated with the IS-like elements that are lacking in *M. thermautotrophicus*. In *M. thermautotrophicus*, amongst the 73 CDS are 18 for CRISPR associated proteins (Cas) (MTH325-327, MTH1076-1088 and MTH1091) of which 17 are lacking in *M. marburgensis* (Table1).

Literature

[1] Liesegang, H., A.K. Kaster, A. Wiezer, M. Goenrich, A. Wollherr, H. Seedorf, G. Gottschalk, and R.K. Thauer, "Complete genome sequence of Methanothermobacter marburgensis, a methanoarchaeon model organism", *J. Bacteriol.*, vol. 192, no. 21, pp. 5850-5851, 2010.

[2] Bingemann, R., A.J. Pierik, and A. Klein, "Influence of the fusion of two subunits of the F_{420}-non-reducing hydrogenase of *Methanococcus voltae* on its biochemical properties", *Arch. Microbiol.*, vol. 174, no. 5, pp. 375-378, 2000.

[3] Randau, L., R. Munch, M.J. Hohn, D. Jahn, and D. Soll, "*Nanoarchaeum equitans* creates functional tRNAs from separate genes for their 5'- and 3'-halves", *Nature*, vol. 433, no. 7025, pp. 537-541, 2005.

[4] Thoma, C., M. Frank, R. Rachel, S. Schmid, D. Nather, G. Wanner, and R. Wirth, "The Mth60 fimbriae of *Methanothermobacter thermoautotrophicus* are functional adhesins", *Environ. Microbiol.*, vol. 10, no. 10, pp. 2785-2795, 2008.

Supplementary Table 1: The 27 protein encoding sequences (CDS) and 7 CDS homolog pairs found in all methanogenic archaea but not in non methanogens

CDS	Annotation
**CDS found in all methanogenic archaea but not in non methanogens**	
MTBMA_c00280 / MTH1441	conserved hypothetical protein (putative kinase)
MTBMA_c01050 / MTH1522	nitrogenase molybdenum-iron protein related (NflD)
MTBMA_c01600 / MTH1574	conserved hypothetical protein (methanogenesis marker protein 8)
MTBMA_c01760 / MTH1590	phosphomannomutase related protein
MTBMA_c02630 / MTH1684	conserved hypothetical protein containing a ferredoxin domain
MTBMA_c04350 / MTH1864	SelD-related protein
MTBMA_c04360 / MTH1865	peptidyl-prolyl cis-trans isomerase related protein
MTBMA_c04370 / MTH1866	conserved hypothetical protein (methanogenesis marker protein 6)
MTBMA_c04390 / MTH1868	conserved hypothetical protein (methanogenesis marker protein 5)
MTBMA_c04410 / MTH1870	conserved hypothetical protein (methanogenesis marker protein 17)
MTBMA_c04420 / MTH1871	NifB-related protein
MTBMA_c06800 / MTH231	methyltransferase related protein (MtxX)
MTBMA_c12680 / MTH873	UDP-N-acetylmuramyl pentapeptide synthase related protein (MurF)
MTBMA_c13710 / MTH987	conserved hypothetical protein
MTBMA_c13960 / MTH1014	predicted metal-binding transcription factor
MTBMA_c15370 / MTH1153	conserved hypothetical protein (putative cobalt transport protein)
MTBMA_c15430 / MTH1159	N^5-methyltetrahydromethanopterin: coenzyme M methyltransferase, subunit A (EC 2.1.1.86) (MtrA)
MTBMA_c15440 / MTH1160	N^5-methyltetrahydromethanopterin: coenzyme M methyltransferase, subunit B (EC 2.1.1.86) (MtrB)
MTBMA_c15450 / MTH1161	N^5-methyltetrahydromethanopterin: coenzyme M methyltransferase, subunit C (EC 2.1.1.86) (MtrC)
MTBMA_c15460 / MTH1162	N^5-methyltetrahydromethanopterin: coenzyme M methyltransferase, subunit D (EC 2.1.1.86) (MtrD)
MTBMA_c15470 / MTH1163	N^5-methyltetrahydromethanopterin: coenzyme M methyltransferase, subunit E (EC 2.1.1.86) (MtrE)
MTBMA_c15500 / MTH1666	methyl coenzyme M reductase I operon associated protein (McrC)
MTBMA_c15570 / MTH1180	methyl-coenzyme M reductase II operon associated protein (MrtC)
MTBMA_c15850 / MTH1207	sulfopyruvate decarboxylase, beta subunit (EC 4.1.1.79) (ComE)
MTBMA_c16830 / MTH1297	F_{420}-reducing hydrogenase, subunit beta (EC 1.12.98.1) (FrhB)
MTBMA_c16840 / MTH1296	F_{420}-reducing hydrogenase, subunit gamma (EC 1.12.98.1) (FrhG)
MTBMA_c17770 / MTH1391	predicted DNA-binding protein

CDS of homolog pairs with at least one being found in all methanogenic archaea but not in non methanogens

MTBMA_c02820 / MTH1703	conserved hypothetical protein (putative membrane protein)
MTBMA_c13650 / MTH980	conserved hypothetical protein (putative membrane protein)
MTBMA_c09140 / MTH126	conserved hypothetical protein
MTBMA_c00070 / MTH1418	conserved hypothetical protein
MTBMA_c15480 / MTH1164	methyl-coenzyme M reductase I, subunit alpha (EC 2.8.4.1) (McrA)
MTBMA_c15120 / MTH1129	methyl-coenzyme M reductase II, subunit alpha (EC 2.8.4.1) (MrtA)
MTBMA_c15490 / MTH1165	methyl-coenzyme M reductase I, subunit gamma (EC 2.8.4.1) (McrG)
MTBMA_c15130 / MTH1130	methyl-coenzyme M reductase II, subunit gamma (EC 2.8.4.1) (MrtG)
MTBMA_c15510 / MTH1167	methyl-coenzyme M reductase I, subunit delta (EC 2.8.4.1) (McrD)
MTBMA_c15140 / MTH1131	methyl-coenzyme M reductase II, subunit delta (EC 2.8.4.1) (MrtD)
MTBMA_c15520 / MTH1168	methyl-coenzyme M reductase I, subunit beta (EC 2.8.4.1) (McrB)
MTBMA_c15150 / MTH1132	methyl-coenzyme M reductase II, subunit beta (EC 2.8.4.1) (MrtB)
MTBMA_c13970 / MTH1015	methyl-coenzyme M reductase, component A2 (AtwA1)
MTBMA_c06010 / MTH454 (and 151)	methyl-coenzyme M reductase, component A2 (AtwA2) homolog

Supplementary Table 2: The 145 CDS in the genome of *Methanothermobacter marburgensis* not found in the genome of *M. thermautotrophicus* with the "most similar CDS method". This method allows the use of a sequence in the comparison only once. Neither the second or third best hits nor CDS homologs within the same organism are revealed. A sequence not in common does therefore not mean that there are no paralogs or orthologs of this sequence within the two organisms. The homologs given in this table were also found by considering significant hits (E value cut-off of e-8) to other CDS in *M. thermautotrophicus* and by considering CDS homologs within *M. marburgensis*. The CDS highlighted with bold numbers are associated with IS-like elements.

CDS	Annotation	Homologs
**Putative gene splitting events: neighboring CDS with second or third best hit**		
MTBMA_c01170	predicted hydrolase (80aa)	MTBMA_c01160 (338aa) + MTBMA_c01170 (80aa) = MTH1534 (424aa)
MTBMA_c02470	predicted transcription initiation factor IIE, alpha subunit (35aa)	MTBMA_c02480 (85aa) + MTBMA_c02470 (35aa) = part of MTH1669 (178aa)
MTBMA_c03150	2-oxopropionate (pyruvate) synthase, subunit D (EC 1.2.7.1) (porD) (81aa)	MTBMA_c03160 (177aa) + MTBMA_c03150 (81aa) = MTH1740 (261aa)
MTBMA_c03360	predicted nucleoside-diphosphate-sugar transferase (EC 2.7.-.-) (141aa)	MTBMA_c03360 (141aa) + MTBMA_c03350 (256aa) = MTH1759 (385aa)
MTBMA_c03720	predicted ATP-dependent helicase (427aa)	MTBMA_c03730 (441aa) + MTBMA_c03720 (427aa) = MTH1802 (862aa)
MTBMA_c04120	predicted hydrolase (93aa)	MTBMA_c04130 (166aa) + MTBMA_c04120 (93aa) = MTH1842 (235aa)
MTBMA_c04190	conserved hypothetical protein (84aa)	MTBMA_c04190 (84aa) + MTBMA_c04180 (259aa) = MTH1848 (365aa)
MTBMA_c05290	30S ribosomal protein S9P (rps9p) (133aa)	MTBMA_c05290 (133aa) + MTBMA_c05280 (140aa) = MTH39 (282aa)
MTBMA_c05530	predicted transporter protein (93aa)	MTBMA_c05530 (93aa) + MTBMA_c05520 (308aa) = part of MTH104 (459aa)
MTBMA_c05720	predicted sensory transduction histidine kinase (49aa)	MTBMA_c05730 (287aa) + MTBMA_c05720 (49aa) = MTH123 (356aa)
MTBMA_c06540	conserved hypothetical protein (140aa)	MTBMA_c06540 (140aa) + MTBMA_c06530 (205aa) = MTH204 (338aa)
MTBMA_c10070	conserved hypothetical protein (41aa)	MTBMA_c10080 (88aa) + MTBMA_c10070 (41aa) = MTH626 (128aa)
MTBMA_c10140	conserved hypothetical protein (171aa)	MTBMA_c10130 (531aa) + MTBMA_c10140 (171aa) = MTH632 (643aa)
MTBMA_c10320	predicted ribonucleoside-diphosphate reductase, large subunit (144aa)	MTBMA_c10330 (573aa) + MTBMA_c10320 (144aa) = MTH652 (703aa)
MTBMA_c10550	cobalto chelatase subunit-likeprotein (604aa)	MTBMA_c10570 (195aa) + MTBMA_c10560 (849aa) + MTBMA_c10550 (604aa) = MTH673 (1708aa)
MTBMA_c10570	cobalto chelatase subunit-like protein (195aa)	MTBMA_c10570 (195aa) + MTBMA_c10560 (849aa) + MTBMA_c10550 (604aa) = MTH673 (1708aa)
MTBMA_c10600	predicted methyltransferase (EC 2.1.1.-) (121aa)	MTBMA_c10610 (165aa) + MTBMA_c10600 (121aa) = MTH675 (266aa)
MTBMA_c10920	2-oxoisovalerate synthase, subunit A (EC 1.2.7.7) (vorA1) (93aa)	MTBMA_c10930 (381aa) + MTBMA_c10920 (93aa) = MTH705 (477aa)
MTBMA_c11570	conserved hypothetical protein (95aa)	MTBMA_c11570 (95aa) + MTBMA_c11560 (154aa) = MTH762 (256aa)
MTBMA_c12130	predicted histone acetyltransferase (EC 2.3.1.48) (188aa)	MTBMA_c12140 (348aa) + MTBMA_c12130 (188aa) = MTH817 (545aa)
MTBMA_c15690	conserved hypothetical protein (71aa)	MTBMA_c15690 (71aa) + MTBMA_c15700 (158aa) = MTH1192 (242aa)
MTBMA_c17860	conserved hypothetical protein (78aa)	MTBMA_c17860 (78aa) + MTBMA_c17870 (174aa) = MTH1400 (261aa)
**Putative gene deletion events: no homologs found in *M. marburgensis* and any other organism used in the comparison**		
MTBMA_c00790	hypothetical protein (54aa)	not found
MTBMA_c00870	hypothetical protein (54aa)	not found
MTBMA_c01150	hypothetical protein (91aa)	not found
MTBMA_c01850	hypothetical protein (56aa)	not found
MTBMA_c03860	hypothetical protein (124aa)	not found
MTBMA_c04320	hypothetical protein (47aa)	not found
MTBMA_c04690	hypothetical protein (57aa)	not found
MTBMA_c05630	hypothetical protein (49aa)	not found
MTBMA_c07340	hypothetical protein (71aa)	not found
MTBMA_c07370	hypothetical protein (146aa)	not found
MTBMA_c07380	hypothetical protein (155aa)	not found
MTBMA_c07460	hypothetical protein (87aa)	not found

MTBMA_c07560	predicted transcriptional regulator (132aa)	not found
MTBMA_c07570	hypothetical protein (85aa)	not found
MTBMA_c07580	hypothetical protein (185aa)	not found
MTBMA_c08150	conserved hypothetical protein (83aa)	fragment of MTH420 (676aa)
MTBMA_c08160	hypothetical protein (76aa)	not found
MTBMA_c08480	cobalto chelatase subunit-likeprotein (109aa)	fragment of MTH456 (859aa)
MTBMA_c08490	cobalto chelatase subunit-likeprotein (51aa)	fragment of MTH456 (859aa)
MTBMA_c08540	hypothetical protein (63aa)	not found
MTBMA_c08560	hypothetical protein (35aa)	fragment of MTH469 (288aa)
MTBMA_c08620	hypothetical protein (102aa)	not found
MTBMA_c08780	hypothetical protein (94aa)	not found
MTBMA_c08880	hypothetical protein (366aa)	not found
MTBMA_c08890	hypothetical protein (166aa)	not found
MTBMA_c09020	hypothetical protein (279aa)	not found
MTBMA_c09970	hypothetical protein (190aa)	not found
MTBMA_c10440	hypothetical protein (53aa)	not found
MTBMA_c10590	hypothetical protein (50aa)	not found
MTBMA_c10980	hypothetical protein (47aa)	not found
MTBMA_c11010	hypothetical protein (61aa)	not found
MTBMA_c11050	hypothetical protein (81aa)	not found
MTBMA_c11640	hypothetical protein (46aa)	not found
MTBMA_c12210	hypothetical protein (82aa)	not found
MTBMA_c14650	hypothetical protein (117aa)	not found
MTBMA_c14660	hypothetical protein (99aa)	not found
MTBMA_c14670	hypothetical protein (53aa)	not found
MTBMA_c14680	hypothetical protein (63aa)	not found
MTBMA_c14730	hypothetical protein (173aa)	not found
MTBMA_c14800	hypothetical protein (62aa)	not found
MTBMA_c15250	hypothetical protein (51aa)	not found
MTBMA_c15940	surface protease related protein (62aa)	not found
MTBMA_c16530	hypothetical protein (109aa)	not found
MTBMA_c17290	hypothetical protein (130aa)	not found

Putative gene duplication events: two or more homologs in *M. marburgensis* where only one is found in *M. thermautotrophicus*

**MTBMA_c00770**	predicted DNA methylase (881aa)	0 relative to MTBMA_c09340 (886aa)
MTBMA_c04680	oligosaccharyl transferase STT3 subunit related protein (706aa)	3e-148 relative to MTBMA_c04670 (694aa)
MTBMA_c04720	conserved hypothetical protein (59aa)	9e-14 relative to MTBMA_c04700 (61aa)
MTBMA_c05450	conserved hypothetical protein (773aa)	0 relative to MTBMA_c10580 (964aa)
MTBMA_c06050	predicted pseudomurein-binding protein (837aa)	8e-92 relative to MTBMA_c07430 (874aa)
MTBMA_c06470	predicted efflux pump protein (455aa)	7e-145 relative to MTBMA_c06460 (458aa)
MTBMA_c07330	predicted nickel responsive regulator (nikR) (142aa)	2e-60 relative to MTBMA_c11340 (140aa)
MTBMA_c07420	predicted pseudomurein-binding protein (611aa)	1e-159 relative to MTBMA_c07430 (874aa)
MTBMA_c07690	predicted glycosyltransferase (EC 2.4.1.-) (382aa)	3e-11 relative to MTBMA_c08450 (363aa)
MTBMA_c08790	predicted sensory transduction histidine kinase (655aa)	4e-65 relative to MTBMA_c06270 (787aa)
**MTBMA_c09340**	predicted adenine-specific DNA methylase (EC 2.1.1.72)(886aa)	0 relative to MTBMA_c00770 (881aa)
MTBMA_c09380	tetratricopeptide repeat domain-containing protein (416aa)	7e-23 relative to MTBMA_c11120 (406aa)
MTBMA_c14190	acetyl-CoA decarbonylase/synthase complex, subunit epsilon (EC 1.2.99.2) (cdhB) (169aa)	8e-35 relative to MTBMA_c02880 (170aa)
MTBMA_c14230	HycB-related protein (128aa)	1e-18 relative to MTBMA_c03120 (162aa)
MTBMA_c14690	predicted type I restriction-modification enzyme, subunit R (EC 3.1.21.3) (hsdR) (962aa)	9e-31 relative to MTBMA_c13260 (1013aa)
MTBMA_c14710	predicted type I restriction-modification enzyme, subunit S (EC 3.1.21.3) (hsdS) (435aa)	2e-12 relative to MTBMA_c13270 (368aa)
MTBMA_c14720	predicted type I restriction-modification enzyme, subunit M (EC 3.1.21.3) (hsdM) (671aa)	4e-36 relative to MTBMA_c13280 (590aa)
MTBMA_c14790	predicted sensory transduction histidine kinase (662aa)	2e-49 relative to MTBMA_c12200 (718aa)
MTBMA_c14910	predicted cobalt transport protein (cbiM) (221aa)	2e-25 relative to MTBMA_c02860 (216aa)

MTBMA_c15870	predicted pseudomurein-binding protein (839aa)	0 relative to MTBMA_c11150 (828aa)
MTBMA_c15890	hypothetical protein (78aa)	6e-20 relative to MTBMA_c11100 (81aa)
MTBMA_c16170	predicted cell surface glycoprotein (590aa)	4e-127 relative to MTBMA_c10780 (683aa)
**MTBMA_c16770**	conserved hypothetical protein (141aa)	5e-60 relative to MTBMA_c17310 (159aa)
**MTBMA_c17310**	conserved hypothetical protein (159aa)	7e-60 relative to MTBMA_c16770 (141aa)

Putative partial gene duplication events: two or more homologs in *M. marburgensis* where only one is found in *M. thermautotrophicus*

MTBMA_c06660	acetyl-coenzyme A synthetase (100aa)	fragment of MTBMA_c01900 (653aa)
MTBMA_c08350	predicted sensory transduction regulatory protein (168aa)	fragment of MTBMA_c08430 (277aa)
MTBMA_c08460	hypothetical protein (71aa)	fragment of MTBMA_c09440 (663aa)
MTBMA_c08470	magnesium chelatase subunit (119aa)	fragment of MTBMA_c09440 (663aa)
MTBMA_c08630	hypothetical protein (287aa)	fragment of MTBMA_c08610 (2549aa)
MTBMA_c08700	hypothetical protein (130aa)	fragmentB relative to MTBMA_c00310 (574aa)/MTBMA_c01890 (524aa)
MTBMA_c08710	hypothetical protein (237aa)	fragmentC relative to MTBMA_c00310 (574aa)/MTBMA_c01890 (524aa)
MTBMA_c08930	predicted tRNA-ribosyltransferase (379aa)	fragment of MTBMA_c06290 (645aa)
MTBMA_c10780	copper chaperone (nitrous oxidase accessory related protein) (683aa)	3e-122 relative to MTBMA_c16170 (590aa) = fragment of MTH716 (1755aa)
MTBMA_c13140	magnesium chelatase subunit related protein (128aa)	fragment of MTBMA_c10550 (604aa)
MTBMA_c14200	acetyl-CoA decarbonylase/synthase complex, alpha subunit (EC 1.2.99.2) (cdhA) (395aa)	fragmentC of MTBMA_c02870 (777aa)
MTBMA_c14210	acetyl-CoA decarbonylase/synthase complex, alpha subunit (EC 1.2.99.2) (cdhA) (311aa)	fragmenB of MTBMA_c02870 (777aa)
MTBMA_c14220	acetyl-CoA decarbonylase/synthase complex, alpha subunit (EC 1.2.99.2) (cdhA) (139aa)	fragmentA of MTBMA_c02870 (777aa)
MTBMA_c14610	predicted cation antiporter (39aa)	fragment of MTBMA_c15390 (318aa)
MTBMA_c15900	hypothetical protein (187aa)	fragment of MTBMA_c11130 (316aa)
MTBMA_c15910	tetratricopeptide repeat domain-containing protein (207aa)	fragment of MTBMA_c11120 (406aa)
**MTBMA_c15920**	conserved hypothetical protein (147aa)	fragment of MTBMA_c11130 (316aa)
MTBMA_c15950	hypothetical protein (65aa)	fragment of MTBMA_c11070 (127aa)
MTBMA_c16180	predicted cell surface glycoprotein (510aa)	fragment of MTBMA_c16170 (590aa)
MTBMA_c16190	predicted cell surface glycoprotein (173aa)	fragment of MTBMA_c16170 (590aa)

Putative lateral gene transfer events: homologs not found in *M. thermautotrophicus* but in other organisms

MTBMA_c00660	conserved hypothetical protein (628aa)	1e-79 relative to ZP_05392980 (505aa) in *Clostridium carboxidivorans* P7
MTBMA_c01240	transposase (163aa)	2e-77 relative to AF1351 (161aa) in *Archaeoglobus fulgidus* DSM 4304
MTBMA_c01250	transposase (165aa)	8e-73 relative to AF1352 (182aa) in *Archaeoglobus fulgidus* DSM 4304
MTBMA_c01530	nitrogenase iron-molybdenum cofactor biosynthesis protein NifX (radical SAM protein) (116aa)	2e-14 relative to MmarC7_0105 (106aa) in *Methanococcus maripaludis* C7
MTBMA_c02220	conserved hypothetical protein (142aa)	fragmentA, 9e-10 relative to AF0002 (175aa) in *Archaeoglobus fulgidus* DSM 4304
MTBMA_c05410	conserved hypothetical protein (100aa)	1e-8 relative to ZP_04878039 (107aa) in *Thermococcus barophilus* MP
MTBMA_c05420	conserved hypothetical protein (122aa)	1e-20 relative to ZP_04878104 (135aa) in *Thermococcus barophilus* MP
MTBMA_c05620	conserved hypothetical protein (50aa)	5e-06 relative to Msm_1607 (69aa) in *Methanobrevibacter smithii* ATCC 35061
MTBMA_c06230	conserved hypothetical protein (292aa)	1e-15 relative to MS0879 (327aa) in *Mannheimia succiniciproducens* MBEL55E
MTBMA_c06240	predicted glycosyltransferase (EC 2.4.1.-) (378aa)	3e-18 relative to YP_001048299 (395aa) in *Methanoculleus marisnigri* JR1
MTBMA_c06870	conserved hypothetical protein (199aa)	4e-50 relative to RCIX2744 (216aa) in *Methanocellales* ord. nov.
MTBMA_c07320	predicted NADH dehydrogenase(196aa)	1e-62 relative to MA0147 (200aa) in *Methanosarcina acetivorans* C2A
MTBMA_c07390	conserved hypothetical protein (624aa)	4e-58 relative to Ava_B0090 (706aa) in *Anabaena variabilis* ATCC 29413
MTBMA_c07550	conserved hypothetical protein (98aa)	2e-07 relative to Mthe_0505 (113aa) in *Methanosaeta thermophila* PT
MTBMA_c07630	predicted glycosyltransferase (EC 2.4.1.-) (389aa)	2e-57 relative to MmarC5_1308 (399aa) in *Methanococcus maripaludis* C5
MTBMA_c07650	predicted methyltransferase (EC 2.1.1.-) (314aa)	2e-14 relative to CA_C3419 (207aa) in *Clostridium acetobutylicum* ATCC 824
MTBMA_c07660	conserved hypothetical protein (214aa)	7e-10 relative to MA2176 (223aa) in *Methanosarcina acetivorans* C2A
MTBMA_c07670	conserved hypothetical protein (216aa)	fragment, 7e-14 relative to ZP_06394863 (514aa) in *Staphylothermus hellenicus* DSM 12710
MTBMA_c07680	conserved hypothetical protein (266aa)	fragment, 3e-10 relative to CAJ70955 (533aa) in *Candidatus Kuenenia stuttgartiensis*
MTBMA_c07710	glycosyltransferase (EC 2.4.1.-) (378aa)	7e-57 relative to Memar_0676 (396aa) in *Methanoculleus marisnigri* JR1
MTBMA_c07740	conserved hypothetical protein (106aa)	fragmentB, 2e-24 relative to CA_P0044 (331aa) in *Clostridium acetobutylicum* ATCC 824
MTBMA_c07750	conserved hypothetical protein (222aa)	fragmentA, 5e-35 relative to CA_P0044 (331aa) in *Clostridium acetobutylicum* ATCC 824
MTBMA_c08580	conserved hypothetical protein (285aa)	2e-20 relative to MA3844 (214aa) in *Methanosarcina acetivorans* C2A

MTBMA_c08590	conserved hypothetical protein (250aa)	7e-08 relative to Mhun_2048 (265aa) in *Methanospirillum hungatei* JF-1
MTBMA_c08610	conserved hypothetical protein (2549aa)	1e-08 relative to Geob_3309 (1946aa) in *Geobacter sp.* FRC-32
MTBMA_c08860	conserved hypothetical protein (81aa)	2e-12 relative to mru_1322 (80aa) in *Methanobrevibacter ruminantium* M1
MTBMA_c08900	conserved hypothetical protein (1407aa)	1e-29 relative to Hbut_1477 (1486aa) in *Hyperthermus butylicus* DSM 5456
MTBMA_c08920	conserved hypothetical protein (364aa)	1e-12 relative to Hbut_1538 (384aa) in *Hyperthermus butylicus* DSM 5456
MTBMA_c09330	conserved hypothetical protein (904aa)	0 relative to YP_003474347 (851aa) in *Thermocrinis albus* DSM 14484
MTBMA_c09350	helicase domain related protein (774aa)	fragmentB, 0 relative to YP_003474345 (1107aa) in *Thermocrinis albus* DSM 14484
MTBMA_c09360	conserved hypothetical protein (294aa)	fragmentA, 4e-88 relative to YP_003474345 (1107aa) in *Thermocrinis albus* DSM 14484
MTBMA_c13340	conserved hypothetical protein (49aa)	7e-09 relative to Msm_0432 (50aa) in *Methanobrevibacter smithii* ATCC
MTBMA_c14700	conserved hypothetical protein (286aa)	4e-47 relative to ZP_05215240 (275aa) in *Mycobacterium avium* subsp. avium ATCC 25291
MTBMA_c14740	conserved hypothetical protein (343aa)	1e-39 relative to MFS40622_0544 (310aa) in *Methanocaldococcus sp.* FS406-22
MTBMA_c17330	conserved hypothetical protein (53aa)	fragmentB, 9e-10 relative to AF0002 (175aa) in *Archaeoglobus fulgidus* DSM 4304

Supplementary Table 3: The 266 CDS in the genome of *Methanothermobacter thermautotrophicus* not found in the genome of *M. marburgensis* with the "most similar CDS method”. This method allows the use of a sequence in the comparison only once. Neither the second or third best hits nor CDS homologues within the same organism are revealed. A sequence not in common does therefore not mean that there are no paralogs or orthologs of this sequence within the two organisms. The homologues given in the table were also found by considering significant hits (E value cut-off of e-8) to other CDS in *M. marburgensis* and by considering homologues within *M. thermautotrophicus*. It is likely that in *M. thermautotrophicus* some of the identified 47 split genes (see below) are in reality the result of sequencing errors since in *M. marburgensis* of the 145 CDS not in common only 22 were traced back to gene splitting events (Supplementary Table 1).

CDS	Annotation	Homologs
**Putative gene splitting events: neighboring CDS with second or third best hit**		
MTH113	arylsulfatase regulatory protein (108aa)	MTH113 (108aa) + MTH114 (262aa) = MTBMA_c05610 (375aa)
MTH216	acetyl-CoA synthetase (43aa)	MTH217 (558aa) + MTH216 (43aa) = MTBMA_c01900 (653aa)
MTH400	energy-converting hydrogenase A, subunit Q1 (ehaQ1) (108aa)	MTH400 (108aa) + MTH401 (337aa) = MTBMA_c08000 (407aa)
MTH425	hypothetical protein (119aa)	MTH425 (119aa) + MTH426 (162aa) = MTBMA_c08210 (294aa)
MTH449	hypothetical protein (120aa)	MTH448 231aa) + MTH449 (120aa) = MTBMA_c08440 (361aa)
MTH458	hypothetical protein (90aa)	MTH457 (592aa) + MTH458 (90aa) + MTH459 (495aa) = MTBMA_c08500 (1203aa)
MTH459	sensory transduction histidine kinase (495aa)	MTH457 (592aa) + MTH458 (90aa) + MTH459 (495aa) = MTBMA_c08500 (1203aa)
MTH485	hypothetical protein (58aa)	MTH488 (204aa) + MTH487 (1157aa) + MTH486 (176aa) + MTH485 (58aa) = MTBMA_c08800 (1596aa)
MTH486	hypothetical protein (176aa)	MTH488 (204aa) + MTH487 (1157aa) + MTH486 (176aa) + MTH485 (58aa) = MTBMA_c08800 (1596aa)
MTH488	hypothetical protein (204aa)	MTH488 (204aa) + MTH487 (1157aa) + MTH486 (176aa) + MTH485 (58aa) = MTBMA_c08800 (1596aa)
MTH513	hypothetical protein (198aa)	MTH513 (198aa) + MTH514 (1045aa) + MTH515 (181aa) = MTBMA_c09040 (1486aa)
MTH515	hypothetical protein (181aa)	MTH513 (198aa) + MTH514 (1045aa) + MTH515 (181aa) = MTBMA_c09040 (1486aa)
MTH521	hypothetical protein (164aa)	MTH521 (164aa) + MTH522 (60aa) = MTBMA_c09100 (227aa)
MTH547	hypothetical protein (252aa)	MTH549 (64aa) + MTH548 (365aa) + MTH547 (252aa) = MTBMA_c09390 (707aa)
MTH549	sensory transduction regulatory protein (64aa)	MTH549 (64aa) + MTH548 (365aa) + MTH547 (252aa) = MTBMA_c09390 (707aa)
MTH568	hypothetical protein (64aa)	MTH568 (64aa) + MTH569 (258aa) = MTBMA_c09550 (317aa)
MTH582	hypothetical protein (110aa)	MTH581 (126aa) + MTH582 (110aa) = MTBMA_c09620 (310aa)
MTH702	acetyl-CoA synthetase related protein (124aa)	MTH701 (425aa) + MTH702 (124aa) = MTBMA_c06650 (548aa)
MTH732	hypothetical protein (96aa)	MTH731 (308aa) + MTH732 (96aa) = MTBMA_c11270 (418aa)
MTH754	hypothetical protein (331aa)	MTH753 (345aa) + MTH754 (331aa) = MTBMA_c11480 (677aa)
MTH839	hypothetical protein (140aa)	MTH838 (307aa) + MTH839 (140aa) = MTBMA_c12360 (410aa)
MTH923	hypothetical protein (101aa)	MTH923 (101aa) + MTH922 (111aa) = MTBMA_c13100 (235aa)
MTH983	hypothetical protein (64aa)	MTH983 (64aa) + MTH984 (341aa) = MTBMA_c13680 (380aa)
MTH988	hypothetical protein (113aa)	MTH988 (113aa) + MTH989 (123aa) = MTBMA_c13720 (227aa)
MTH1117	phosphoenolpyruvate synthase (62aa)	MTH1118 (684aa) + MTH1117 (62aa) = MTBMA_c15010 (756aa)
MTH1257	hypothetical protein (63aa)	MTH1257 (63aa) + MTH1258 (567aa) = MTBMA_c16450 (642aa)
MTH1278	hypothetical protein (104aa)	MTH1278 (104aa) + MTH1279 (234aa) = MTBMA_c16640 (333aa)
MTH1340	hypothetical protein (113aa)	MTH1341 (62aa) + MTH1340 (113aa) = MTBMA_c17220 (167aa)
MTH1384	replication factor A related protein (181aa)	MTH1385 (622aa) + MTH1384 (181aa) = MTBMA_c17710 (792aa)
MTH1420	hypothetical protein (121aa)	MTH1419 (432aa) + MTH1420 (121aa) = MTBMA_c00080 (502aa)
MTH1429	bacitracin resistance protein (106aa)	MTH1429 (106aa) + MTH1428 (181aa) = MTBMA_c00160 (276aa)
MTH1450	hypothetical protein (205aa)	MTH1450 (205aa) + MTH1451 (236aa) = MTBMA_c00360 (454aa)
MTH1466	hypothetical protein (128aa)	MTH1466 (128aa) + MTH1465 (123aa) = MTBMA_c00510 (260aa)
MTH1469	molybdenum ABC transporter, ATP-binding protein (73aa)	MTH1470 (131aa) + MTH1469 (73aa) = MTBMA_c00540 (223aa)
MTH1472	O-antigen transporter-like protein (132aa)	MTH1472 (132aa) + MTH1471 (366aa) = MTBMA_c00550 (518aa)
MTH1479	metal-dependent phosphoesterase (probable isoleucyl-tRNA synthetase) (85aa)	MTH1478 (154aa) + MTH1479 (85aa) = MTBMA_c00610 (220aa)

MTH1504	hypothetical protein (114aa)	MTH1504 (114aa) + MTH1503 (114aa) = MTBMA_c00890 (223aa)
MTH1604	acetyl-CoA synthetase (249aa)	MTH1603 (400aa) + MTH1604 (249aa) = MTBMA_c01900 (653aa)
MTH1647	hypothetical protein (104aa)	MTH1646 (237aa) + MTH1647 (104aa) = MTBMA_c02300 (349aa)
MTH1688	hypothetical protein (58aa)	MTH1688 (58aa) + MTH1687 (151aa) = MTBMA_c02660 (213aa)
MTH1750	hypothetical protein (59aa)	MTH1750 (59aa) + MTH1749 (348aa) = MTBMA_c03250 (427aa)
MTH1778	hypothetical protein (73aa)	MTH1779 (159aa) + MTH1778 (73aa) = MTBMA_c03540 (241aa)
MTH1782	hypothetical protein (94aa)	MTH1782 (94aa) + MTH1783 (154aa) = MTBMA_c03560 (276aa)
MTH1786	hypothetical protein (101aa)	MTH1786 (101aa) + MTH1785 (353aa) = MTBMA_c03580 (453aa)
MTH1798	hypothetical protein (65aa)	MTH1799 (86aa) + MTH1798 (65aa) = MTBMA_c03700 (158aa)
MTH1805	hypothetical protein (111aa)	MTH1805 (111aa) + MTH1804 (143aa) = MTBMA_c03750 (314aa)
MTH1845	hypothetical protein (42aa)	MTH1844 (248aa) + MTH1845 (42aa) = MTBMA_c04150 (295aa)

Putative gene deletion events: no homologs found in *M. thermautotrophicus* and any other organism used in the comparison

MTH58	hypothetical protein (85aa)	not found
MTH59	hypothetical protein (193aa)	not found
MTH60	fimbrin (176aa)	not found [4]
MTH64	hypothetical protein (92aa)	not found
MTH69	hypothetical protein (85aa)	not found
MTH85	hypothetical protein (41aa)	not found
MTH88	hypothetical protein (35aa)	not found
MTH89	hypothetical protein (59aa)	not found
MTH90	hypothetical protein (130aa)	not found
MTH92	hypothetical protein (70aa)	not found
MTH98	hypothetical protein (41aa)	not found
MTH99	hypothetical protein (59aa)	not found
MTH110	hypothetical protein (82aa)	not found
MTH186	hypothetical protein (60aa)	not found
MTH189	hypothetical protein (60aa)	not found
MTH197	hypothetical protein (62aa)	not found
MTH202	hypothetical protein (74aa)	not found
MTH206	hypothetical protein (79aa)	not found
MTH283	protein kinase (317aa)	not found
MTH285	hypothetical protein (351aa)	not found
MTH286	hypothetical protein (146aa)	not found
MTH288	hypothetical protein (337aa)	not found
MTH289	hypothetical protein (499aa)	not found
MTH294	hypothetical protein (154aa)	not found
MTH295	hypothetical protein (114aa)	not found
MTH300	hypothetical protein (46aa)	not found
MTH302	hypothetical protein (181aa)	not found
MTH319	hypothetical protein (53aa)	not found
MTH320	hypothetical protein (110aa)	not found
MTH322	hypothetical protein (36aa)	not found
MTH350	hypothetical protein (196aa)	not found
MTH353	hypothetical protein (212aa)	not found
MTH355	hypothetical protein (70aa)	not found
MTH363	hypothetical protein (273aa)	not found
MTH364	hypothetical protein (75aa)	not found
MTH366	hypothetical protein (49aa)	not found
MTH407	hypothetical protein (92aa)	not found
MTH410	hypothetical protein (83aa)	not found
MTH452	hypothetical protein (184aa)	not found

MTH461	hypothetical protein (141aa)	not found
MTH462	hypothetical protein (265aa)	not found
MTH467	hypothetical protein (53aa)	not found
MTH493	hypothetical protein (187aa)	not found
MTH565	hypothetical protein (66aa)	not found
MTH575	hypothetical protein (54aa)	not found
MTH629	hypothetical protein (57aa)	not found
MTH640	hypothetical protein (56aa)	not found
MTH642	hypothetical protein (65aa)	not found
MTH1102	hypothetical protein (86aa)	not found
MTH1103	hypothetical protein (63aa)	not found
MTH1169	hypothetical protein (48aa)	not found
MTH1209	hypothetical protein (104aa)	not found
MTH1266	hypothetical protein (79aa)	not found
MTH1333	hypothetical protein (56aa)	not found
MTH1411	hypothetical protein (50aa)	not found
MTH1517	hypothetical protein (85aa)	not found
MTH1518	hypothetical protein (133aa)	not found
MTH1532	hypothetical protein (194aa)	not found
MTH1637	hypothetical protein (58aa)	not found
MTH1638	hypothetical protein (155aa)	not found
MTH1650	hypothetical protein (73aa)	not found
MTH1667	hypothetical protein (46aa)	not found
MTH1771	hypothetical protein (53aa)	not found
MTH1775	hypothetical protein (179aa)	not found
MTH1800	hypothetical protein (100aa)	not found
MTH1851	hypothetical protein (63aa)	not found
MTH1899	hypothetical protein (57aa)	not found

Putative gene duplication events: two or more homologs in *M. thermautotrophicus* where only one is found in *M. marburgensis*

MTH67	hypothetical protein (60aa)	4e-29 relative to MTH82 (60aa)
MTH68	TPR repeat-containing protein (228aa)	1e-72 relative to MTH72 (403aa)
MTH82	hypothetical protein (60aa)	4e-29 relative to MTH67 (60aa)
MTH87	surface protease related protein (840aa)	0 relative to MTH75 (837aa)
MTH332	LPS biosynthesis RfbU related protein (400aa)	6e-36 relative to MTH173 (382aa)
MTH333	GDP-D-mannose dehydratase (348aa)	4e-21 relative to MTH1789 (336aa)
MTH334	perosamine synthetase (363aa)	2e-79 relative to MTH1188 (360aa)
MTH335	galactosyl-transferase RfpB related protein (373aa)	9e-22 relative to MTH450 (411aa)
MTH338	LPS biosynthesis RfbU related protein (350aa)	4e-18 relative to MTH450 (411aa)
MTH342	succinoglycan biosynthesis transport protein (477aa)	1e-54 relative to MTH379 (475aa)
MTH351	magnesium chelatase subunit (1561aa)	0 relative to MTH673 (1708aa)
MTH356	sensory transduction histidine kinase (567aa)	4e-42 relative to MTH174 (785aa)
MTH357	Conserved hypothetical protein (735aa)	5e-74 relative to MTH412 (583aa)
MTH361	teichoic acid biosynthesis protein RodC related protein (369aa)	5e-1082 relative to MTH365 (409aa)
MTH365	teichoic acid biosynthesis protein RodC related protein (409aa)	5e-108 relative to MTH361 (369aa)
MTH370	LPS biosynthesis RfbU related protein (395aa)	4e-17 relative to MTH450 (411aa)
MTH373	dTDP-glucose 4,6-dehydratase related protein (334aa)	1e-24 relative to MTH1789 (336aa)
MTH375	UDP-glucose 4-epimerase related protein (332aa)	1e-38 relative to MTH380 (316aa)
MTH420	hypothetical protein (676aa)	1e-65 relative to MTH1906 (711aa)
MTH451	magnesium chelatase subunit ChlI (591aa)	9e-105 relative to MTH556 (395aa)
MTH454	methyl coenzyme M reductase system, component A2-like protein(480aa)	4e-35 relative to MTH1015 (540aa)
MTH464	hypothetical protein (153aa)	6e-26 relative to MTH408 (139aa)

MTH503	hypothetical protein (107aa)	1e-48 relative to MTH1836 (115aa)
MTH571	nitrogenase reductase related protein (263aa)	1e-23 relative to MTH1120 (273aa)
MTH714	magnesium chelatase subunit (1787aa)	0 relative to MTH673 (1708aa)
MTH715	hypothetical protein (317aa)	8e-59 relative to MTH717 (446aa)
MTH717	hypothetical protein (446aa)	7e-55 relative to MTH715 (317aa)
MTH928	cobalamin biosynthesis protein N (1668aa)	0 relative to MTH673 (1708aa)
MTH1172	cation transporter (407aa)	2e-32 relative to MTH1893 (298aa)
MTH1173	cell division inhibitor related protein (298aa)	3e-21 relative to MTH1174 (291aa)
MTH1174	cell division inhibitor related protein (291aa)	7e-26 relative to MTH1173 (298aa)
MTH1331	hypothetical protein (121aa)	6e-61 relative to MTH1652 (121aa)
MTH1346	hypothetical protein (933aa)	0 relative to MTH674 (966aa)
MTH1364	hypothetical protein (236aa)	3e-25 relative to MTH526 (228aa)
MTH1653	hypothetical protein (321aa)	4e-159 relative to MTH1330 (319aa)
MTH1780	stomatin-like protein (297aa)	1e-36 relative to MTH692 (318aa)
MTH1825	cell surface glycoprotein (674aa)	3e-11 relative to MTH719 (574aa)
MTH1898	oligosaccharyl transferase (672aa)	9e-65 relative to MTH1906 (711aa)

Putative partial gene duplication events: two or more homologs in *M. thermautotrophicus* where only one is found in *M. marburgensis*

MTH54	hypothetical protein (212aa)	fragmentD of MTH674 (966aa)/MTH1346 (933aa)
MTH55	hypothetical protein (377aa)	fragmentC of MTH674 (966aa)/MTH1346 (933aa)
MTH56	hypothetical protein (165aa)	fragmentB of MTH674 (966aa)/MTH1346 (933aa)
MTH57	hypothetical protein (127aa)	fragmentA of MTH674 (966aa)/MTH1346 (933aa)
MTH65	hypothetical protein (127aa)	fragment of MTH75 (837aa)/MTH87 (840aa)
MTH66	hypothetical protein (73aa)	fragment of MTH75 (837aa)/MTH87 (840aa)
MTH70	hypothetical protein (61aa)	fragment of MTH75 (837aa)/MTH87 (840aa)
MTH71	hypothetical protein (88aa)	fragment of MTH75 (837aa)/MTH87 (840aa)
MTH84	hypothetical protein (200aa)	fragment of MTH73 (316aa)
MTH91	hypothetical protein (75aa)	fragment of MTH1653 (321aa)/MTH1330 (319aa)
MTH94	hypothetical protein (90aa)	fragment of MTH1653 (321aa)/MTH1330 (319aa)
MTH96	hypothetical protein (189aa)	fragment of MTH1653 (321aa)/MTH1330 (319aa)
MTH97	hypothetical protein (92aa)	fragment of MTH1653 (321aa)/MTH1330 (319aa)
MTH282	hypothetical protein (308aa)	fragment of MTH510 (473aa)
MTH297	DNA-dependent RNA polymerase, subunit A' (133aa)	fragmentA of MTH1051 (870aa)
MTH298	DNA-dependent RNA polymerase, subunit A' (89aa)	fragmentB of MTH1051 (870aa)
MTH299	DNA-dependent RNA polymerase, subunit A' (664aa)	fragmentC of MTH1051 (870aa)
MTH301	hypothetical protein (172aa)	fragment of MTH75 (837aa)/MTH87 (840aa)
MTH317	cobalamin biosynthesis protein N (826aa)	fragmentB of MTH1363 (1329aa)
MTH318	cobalamin biosynthesis protein N (533aa)	fragmentA of MTH1363 (1329aa)
MTH359	hypothetical protein (534aa)	fragment of MTH357 (735aa)
MTH360	sensory transduction histidine kinase (700aa)	fragment of MTH174 (785aa)
MTH367	O-antigen transporter (289aa)	fragment of MTH347 (420aa)
MTH374	dolichyl-phosphate mannose synthase related protein (291aa)	fragment of MTH1392 (564aa)
MTH377	dolichyl-phosphate mannose synthase related protein (248aa)	fragment of MTH1392 (564aa)
MTH456	magnesium chelatase subunit (859aa)	fragment of MTH1363 (1329aa)
MTH481	cation-transporting ATPase (404aa)	fragment of MTH1001 (844aa)
MTH482	cation-transporting ATPase (90aa)	fragment of MTH1001 (844aa)
MTH496	endonuclease III-like protein (175aa)	fragment of MTH764 (233aa)
MTH510	hypothetical protein (473aa)	fragment of MTH282 (308aa)
MTH531	UDP-N-acetylmuramyl tripeptide synthetase related protein (312aa)	fragment of MTH530 (682aa)
MTH555	magnesium chelatase subunit (182aa)	fragmentB of MTH451 (591aa)
MTH718	hypothetical protein (73aa)	fragment of MTH720 (388aa)
MTH719	cell surface glycoprotein (s-layer protein) (574aa)	fragment of MTH716 (1755aa)

MTH910	hypothetical protein (216aa)	fragment of MTH977 (394aa)
MTH911	probable surface protein (698aa)	fragment of MTH716 (1755aa)
MTH913	hypothetical protein (433aa)	fragment of MTH674 (966aa)/MTH1346 (933aa)
MTH914	hypothetical protein (421aa)	fragment of MTH674 (966aa)/MTH1346 (933aa)
MTH977	endo-1,4-beta-glucanase related protein (394aa)	fragment of MTH716 (1755aa)
MTH1175	hypothetical protein (124aa)	fragment of MTH1172 (407aa)
MTH1332	hypothetical protein (189aa)	fragment of MTH1653 (321aa)/MTH1330 (319aa)
MTH1651	hypothetical protein (172aa)	fragment of MTH1653 (321aa)/MTH1330 (319aa)

Putative lateral gene transfer events: homologs not found in *M. marburgensis* but in other organisms

MTH63	4-carboxymuconolactone decarboxylase (EC 4.1.1.44) (114aa)	3e-11 relative to MM_0918(165aa) in *Methanosarcina mazei* Go1
MTH196	ATPase (458aa)	3e-178 relative to ZP_04879563 (464aa) in *Thermococcus sp.* AM4
MTH276	hypothetical protein (128aa)	fragment, 6e-08 relative to MCP_0354 (588aa) in *Methanocella paludicola* SANAE
MTH284	ATP-dependent Clp protease regulatory subunit (616aa)	4e-76 relative to ZP_06025640 (616aa) in *Fusobacterium periodonticum* ATCC 33693
MTH287	anaerobic ribonucleoside-triphosphate reductase activating protein (237aa)	2e-34 relative to CLD_3564 (201aa) in *Clostridium botulinum* B1 str. Okra
MTH293	conserved hypothetical protein (177aa)	9e-56 relative to ZP_05030251 (171aa) in *Microcoleus chthonoplastes* PCC 7420
MTH296	conserved hypothetical protein (419aa)	2e-54 relative to mru_0788 (317aa) in *Methanobrevibacter ruminantium* M1
MTH303	conserved hypothetical protein (345aa)	9e-14 relative to CBH37168 (506aa) in uncultured archaeon
MTH306	conserved hypothetical protein (339aa)	1e-28 relative to ZP_03607154 (352aa) in *Methanobrevibacter smithii* DSM 2375
MTH315	histidine kinase-like ATPases (254aa)	5e-80 relative to psiM100p09 (252aa) in Methanothermobacter phage psiM100
MTH316	conserved hypothetical protein (114aa)	1e-23 relative to psiM100p10 (107aa) in Methanothermobacter phage psiM100
MTH323	conserved hypothetical protein (300aa)	5e-93 relative to Tlet_1201 (300aa) in *Thermotoga lettingae* TMO
MTH324	conserved hypothetical protein (113aa)	1e-29 relative to Tlet_1200 (122aa) in *Thermotoga lettingae* TMO
MTH325	CRISPR-associated RAMP protein, Cmr4 family (287aa)	5e-82 relative to Tlet_1199 (286aa) in *Thermotoga lettingae* TMO
MTH326	CRISPR-associated protein, Csx11 family (957aa)	0 relative to Tlet_1205 (1218aa) in *Thermotoga lettingae* TMO
MTH327	predicted CRISPR-associated RAMP protein (261aa)	1e-63 relative to Tlet_1203 (282aa) in *Thermotoga lettingae* TMO
MTH328	CRISPR-associated RAMP protein, Cmr1 family (251aa)	3e-26 relative to Tlet_1202 (335aa) in *Thermotoga lettingae* TMO
MTH329	conserved hypothetical protein (184aa)	2e-29 relative to mru_1525 (258aa) in *Methanobrevibacter ruminantium* M1
MTH336	conserved hypothetical protein (173aa)	3e-10 relative to ZP_06192739 (173aa) in *Serratia odorifera* 4Rx13
MTH337	conserved hypothetical protein (301aa)	6e-51 relative to CHY_2660 (326aa) in *Carboxydothermus hydrogenoformans* Z-2901
MTH340	polysaccharide pyruvyl transferase CsaB (400aa)	1e-62 relative to Memar_0956 (407aa) in *Methanoculleus marisnigri* JR1
MTH341	coenzyme F420-reducing hydrogenase, beta subunit homolog (406aa)	7e-76 relative to DET0214 (428aa) in *Dehalococcoides ethenogenes* 195
MTH345	pyruvate formate-lyase 2 activating enzyme (288aa)	8e-44 relative to ZP_02993369 (300aa) in *Clostridium sporogenes* ATCC 15579
MTH346	formate acetyltransferase 2 (pyruvate formate lyase) (642aa)	2e-105 relative to DSY3016 (626aa) in *Desulfitobacterium hafniense* Y51
MTH352	conserved hypothetical protein (131aa)	4e-30 relative to CBH36703 (122aa) in uncultured archaeon
MTH354	conserved hypothetical protein (297aa)	2e-31 relative to ZP_04929371 (359aa) in *Pseudomonas aeruginosa* C3719
MTH358	gluthatione-regulated K^+/H^+ antiporter (512aa)	1e-115relative to Dole_0855 (674aa) in *Desulfococcus oleovorans* Hxd3
MTH362	capsular polysaccharide biosynthesis protein (405aa)	2e-36 relative to MJ1059 (406aa) in *Methanocaldococcus jannaschii* DSM 2661
MTH368	glycerol-3-phosphate dehydrogenase (NAD) (321aa)	1e-46 relative to ZP_03607422 (321aa) in *Methanobrevibacter smithii* DSM 2375
MTH369	UDP-N-acetylglucosamine pyrophosphorylase related protein (231aa)	2e-53 relative to DvMF_1841 (225aa) in *Desulfovibrio vulgaris* str. 'Miyazaki F'
MTH371	conserved hypothetical protein fragmentA (245aa)	5e-32 relative to Msp_0616 (540aa) in *Methanosphaera stadtmanae* DSM 3091
MTH372	conserved hypothetical protein fragmentB (287aa)	7e-16 relative to Msp_0616 (540aa) in *Methanosphaera stadtmanae* DSM 3091
MTH453	cobalt transport protein (252aa)	6e-16 relative to Dtox_2654 (231aa) in *Desulfotomaculum acetoxidans* DSM 771
MTH455	hypothetical protein (261aa)	fragment, 2e-15 relative to Daud_1210 (1210aa) in *Candidatus Desulforudis audaxviator* MP104C
MTH495	modification methyltransferase, cytosine-specific (413aa)	8e-106 relative to MM_0278 (423aa) in *Methanosarcina mazei* Gö1
MTH497	Superfamily II DNA and RNA helicase (1235aa)	0 relative to MM_0276 (1275aa) in *Methanosarcina mazei* Gö1
MTH498	conserved hypothetical protein (578aa)	1e-114 relative to MM_0275 (600aa) in *Methanosarcina mazei* Gö1
MTH499	conserved hypothetical protein (971aa)	3e-97 relative to MM_0273 (1009aa) in *Methanosarcina mazei* Gö1
MTH501	5-methylcytosine-specific restriction enzyme McrB related protein (546aa)	fragment, 2e-82 relative to mru_0062 (922aa) in *Methanobrevibacter ruminantium* M1
MTH502	conserved hypothetical protein (567aa)	2e-78 relative to mru_0063 (547aa) in *Methanobrevibacter ruminantium* M1
MTH551	DNA helicase II related protein (853aa)	7e-55 relative to ZP_01877893 (621aa) in *Roseovarius sp.* TM1035
MTH864	transcriptional regulator, ArsR family (250aa)	5e-62 relative to RCIX2193 (250aa) in *Methanocella paludicula*

MTH865	conserved hypothetical protein (81aa)	5e-24 relative to Maeo_0093 (79aa) in *Methanococcus aeolicus* Nankai-3
MTH909	conserved hypothetical protein (182aa)	2e-16 relative to ZP_04876511 (164aa) in *Thermococcus barophilus* MP
MTH912	predicted metallo-beta-lactamase (521aa)	2e-90 relative to CENSYa_0006 (496aa) in *Cenarchaeum symbiosum* A
MTH927	ferredoxin (77aa)	8e-09 relative to Msm_0784 (63aa) in *Methanobrevibacter smithii* ATCC 35061
MTH1076	CRISPR-associated protein TIGR02710 family (400aa)	3e-51 relative to mru_1188 (429aa) in *Methanobrevibacter ruminantium* M1
MTH1077	CRISPR-associated RAMP protein Csm5 family (75aa)	fragmentB, 1e-06 relative to mru_1179 (400aa) in *Methanobrevibacter ruminantium* M1
MTH1078	CRISPR-associated RAMP protein, Csm5 family (286aa)	fragmentA, 2e-09 relative to mru_1179 (400aa) in *Methanobrevibacter ruminantium* M1
MTH1079	CRISPR-associated RAMP protein, Csm4 family (302aa)	1e-42 relative to mru_1180 (331aa) in *Methanobrevibacter ruminantium* M1
MTH1080	CRISPR-associated RAMP protein, Csm3 family (245aa)	2e-53 relative to mru_1181 (255aa) in *Methanobrevibacter ruminantium* M1
MTH1081	CRISPR-associated protein, Csm2 family (140aa)	5e-18 relative to mru_1182 (143aa) in *Methanobrevibacter ruminantium* M1
MTH1082	CRISPR-associated protein, Csm1 family (822aa)	5e-173 relative to mru_1183 (885aa) in *Methanobrevibacter ruminantium* M1
MTH1083	CRISPR-associated protein Cas2 (91aa)	1e-19 relative to mru_0799 (88aa) in *Methanobrevibacter ruminantium* M1
MTH1084	CRISPR-associated protein Cas1 (334aa)	2e-125 relative to mru_0798 (322aa) in *Methanobrevibacter ruminantium* M1
MTH1085	CRISPR-associated protein Cas4 (175aa)	2e-35 relative to mru_0797 (168aa) in *Methanobrevibacter ruminantium* M1
MTH1086	CRISPR-associated protein Cas3 (830aa)	2e-171 relative to ZP_04880468 (868aa) in *Thermococcus sp.* AM4
MTH1087	CRISPR-associated protein Cas5 (269aa)	1e-53 relative to ZP_04880232 (275aa) in *Thermococcus sp.* AM4
MTH1088	CRISPR-associated autoregulator DevR (391aa)	3e-120 relative to ZP_04880262 (393aa) in *Thermococcus sp.* AM4
MTH1089	conserved hypothetical protein (105aa)	5e-17 relative to ZP_04880306 (100aa) in *Thermococcus sp.* AM4
MTH1090	conserved hypothetical protein (598aa)	7e-83 relative to ZP_04880496 (636aa) in *Thermococcus sp.* AM4
MTH1091	CRISPR-associated protein Cas6 (154aa)	3e-31 relative to mru_0792 (243aa) in *Methanobrevibacter ruminantium* M1
MTH1176	predicted archeal iron-sulfur cluster carrier protein ApbC/Npb35 (276aa)	6e-71 relative to Msp_0283 (285aa) in *Methanosphaera stadtmanae* DSM 3091
MTH1177	dinitrogenase iron-molybdenum cofactor biosynthesis protein (116aa)	4e-17 relative to Mbar_A1833 (125aa) in *Methanosarcina barkeri* str. Fusaro
MTH1365	4Fe-4S ferredoxin iron-sulfur binding domain-containing protein (106aa)	3e-36 relative to Mthe_1417 (102aa) in *Methanosaeta thermophila* PT
MTH1366	conserved hypothetical protein (68aa)	5e-06 relative to Ferp_0201 (97aa) in *Ferroglobus placidus* DSM 10642
MTH1625	conserved hypothetical protein (86aa)	9e-09 relative to mru_0389 (81aa) in *Methanobrevibacter ruminantium* M1
MTH1716	hypothetical protein (229aa)	fragment, 2e-19 relative to ZP_04878004 (813aa) in *Thermococcus barophilus* MP
MTH1717	conserved hypothetical protein (557aa)	3e-95 relative to ZP_04878004 (813aa) in *Thermococcus barophilus* MP
MTH1719	conserved hypothetical protein (74aa)	fragment, 4e-06 relative to CBH38353 (895aa) in uncultured archaeon
MTH1725	conserved hypothetical protein (92aa)	6e-23 relative to CBH39270 (89aa) in uncultured archaeon
MTH1892	conserved hypothetical protein (168aa)	4e-15 relative to CBH38812 (165aa) in uncultured archaeon
